# Systematic literature review of the epidemiology of glyphosate and neurological outcomes

**DOI:** 10.1007/s00420-022-01878-0

**Published:** 2022-05-23

**Authors:** Ellen T. Chang, Nnaemeka U. Odo, John F. Acquavella

**Affiliations:** 1 Center for Health Sciences, Exponent, Inc., 149 Commonwealth Dr, Menlo Park, CA 94025 USA; 2 Department of Epidemiology and Biostatistics, University of California, San Francisco, CA USA; 3 Center for Health Sciences, Exponent, Inc., Oakland, CA, USA; 4 Department of Clinical Epidemiology, University of Aarhus, Aarhus, Denmark

**Keywords:** Glyphosate, Neurotoxicity, Nervous system diseases, Epidemiology, Systematic review

## Abstract

**Purpose:**

Human health risk assessments of glyphosate have focused on animal toxicology data for determining neurotoxic potential. Human epidemiological studies have not yet been systematically reviewed for glyphosate neurotoxicity hazard identification. The objective of this systematic literature review was to summarize the available epidemiology of glyphosate exposure and neurological outcomes in humans.

**Methods:**

As of December 2021, 25 eligible epidemiological studies of glyphosate exposure and neurological endpoints were identified and assessed for five quality dimensions using guidance from the U.S. Environmental Protection Agency. Studies that assessed personal use of glyphosate were prioritized, whereas those assessing indirect exposure (other than personal use) were rated as low quality, since biomonitoring data indicate that indirect metrics of glyphosate exposure almost always equate to non-detectable glyphosate doses.

**Results:**

Overall, the scientific evidence on glyphosate and neurotoxicity in humans is sparse and methodologically limited, based on nine included epidemiological studies of neurodegenerative outcomes (two high quality), five studies of neurobehavioral outcomes (two high quality), six studies of neurodevelopmental outcomes (none high quality), and five studies of other and mixed neurological outcomes (one high quality). The five high-quality studies showed no association between glyphosate use and risk of depression, Parkinson disease, or peripheral nerve conduction velocity. Results were mixed among the eight moderate-quality studies, which did not demonstrate consistent associations with any neurological endpoints or categories. Low-quality studies were considered uninformative about possible neurotoxic effects due primarily to questionable assessments of indirect exposure.

**Conclusions:**

No association has been demonstrated between glyphosate and any neurological outcomes in humans. To move the state of science forward, epidemiological studies should focus on scenarios involving direct and frequent use of glyphosate while collecting information on validated health outcomes, concomitant agricultural exposures, and relevant personal characteristics.

**Supplementary Information:**

The online version contains supplementary material available at 10.1007/s00420-022-01878-0.

## Introduction

Glyphosate is a non-selective phosphonomethyl amino acid herbicide that is widely used to control weeds in agriculture, forestry, and lawn and garden care. Following glyphosate’s initial registration in 1974, the U.S. Environmental Protection Agency (EPA) has routinely reviewed and reassessed the safety and uses of glyphosate, in part by implementing the registration review process every 15 years (U.S. EPA 2022). In January 2020, U.S. EPA released its latest interim decision for registration review of glyphosate, in which the Agency concluded that there are no risks of concern to human health when glyphosate is used in accordance with its current label. Likewise, the European Food Safety Authority (EFSA) and European Chemicals Agency recently began their renewal reassessments for glyphosate (EFSA 2021).

While substantial scientific and public attention has been focused on the carcinogenic potential of glyphosate (U.S. EPA 2017b), neurological outcomes have been less frequently studied. In its most recent draft human health risk assessment in support of registration review for glyphosate, U.S. EPA concluded: “There was no evidence that glyphosate is neurotoxic” (U.S. EPA 2017a). This conclusion was based primarily on a limited number of acceptable toxicity studies in animals.

Nevertheless, neurological outcomes spanning the age spectrum, including neurodevelopmental disorders and chronic neurodegenerative diseases, have become increasingly prominent issues in human health risk assessments of pesticides, including glyphosate. Uncertainties and limitations in the extrapolation of data from laboratory animals to humans make it important to consider whether informative epidemiological data exist and, if so, to incorporate epidemiological findings into the process of human health risk assessment (Déglin et al. 2021). To date, however, a thorough review of the epidemiological evidence on glyphosate and neurotoxicity has been lacking. Therefore, we undertook this systematic literature review to identify, assess, and summarize the current state of the epidemiology on glyphosate exposure and neurological outcomes in humans.

## Methods

### Literature search

This systematic literature review was conducted in accordance with procedures described in the Preferred Reporting Items for Systematic Reviews and Meta-Analyses (PRISMA) 2020 statement (Page et al. 2021). Following PRISMA guidance, we developed a Population–Exposure–Comparator–Outcome–Study Design (PECOS) statement to delineate the objectives of our review, as follows:

- Population: humans
- Exposure: glyphosate exposure
- Comparator: absence of glyphosate exposure
- Outcome: chronic neurological conditions, including central and peripheral nervous system disorders, excluding acute poisoning and intoxication events, acute nonspecific neurological symptoms (e.g., headache, dizziness), and nervous system neoplasms
- Study design: comparative epidemiological studies, including cross-sectional, case–control, and cohort studies, excluding case reports and case series.

Studies considered eligible for inclusion in our literature review were human epidemiological studies that reported a measure of association (or data sufficient to calculate a measure of association) between any chronic neurological condition, as described above, and glyphosate exposure, including direct exposure via personal use (i.e., spraying or mixing) of glyphosate, as well as possible indirect exposure via routes other than personal use. Measures of association included relative risks (e.g., odds ratios, risk ratios, hazard ratios), risk differences, correlation coefficients, etc. We excluded animal, in vitro, and mechanistic studies, because this review is limited to human epidemiology. We also excluded case reports and case series, because they lack comparators, and thus cannot estimate exposure–outcome associations, and reviews, editorials, commentaries, letters, and other articles that did not report original data, although we examined the reference lists of these sources to identify additional potentially relevant studies.

Guided by the PECOS statement, we developed the following search string for use in PubMed, including a broad list of exposure terms to capture studies that reported results for glyphosate in the text but not the title, abstract, or keywords:

(glyphosate OR pesticide* OR herbicide*)

#### AND

(neurotoxic* [tiab] OR neurodevelopment* [tiab] OR neurobehavior* [tiab] OR neurobehaviour* [tiab] OR neurologic* [tiab] OR attention [tiab] OR cognit* [tiab] OR "developmental disability" [tiab] OR social [tiab] OR intelligence [tiab] OR memory [tiab] OR learning [tiab] OR brain [tiab] OR psychomotor [tiab] OR behavior* [tiab] OR behaviour* [tiab] OR "Nervous System"[tiab] OR parkinson* [tiab] OR tremor [tiab] OR “movement disorder” [tiab] OR mental [tiab] OR emotion* [tiab] OR cognitive [tiab] OR cognition [tiab] OR dementia [tiab] OR neuronal [tiab] OR neuropathy [tiab] OR motor [tiab] OR sensory [tiab] OR neurodegen* [tiab] OR depression [tiab] OR mood [tiab] OR personality [tiab] OR IQ [tiab] OR autis* [tiab] OR “amyotrophic lateral sclerosis” [tiab] OR Alzheimer* [tiab] OR “congenital anomaly” [tiab] OR “congenital anomalies” [tiab] OR “congenital disorder” [tiab] OR “congenital disorders” [tiab] OR “birth defect” [tiab] or “birth defects” [tiab] OR “neural tube defect” [tiab] OR “neural tube defects” [tiab] OR “spina bifida” [tiab] OR “anencephaly” [tiab])

NOT

(poisoning [tiab] OR intoxication [tiab] OR cancer [tiab] OR lymphoma [tiab] OR leukemia [tiab] OR tumor [tiab] OR malignan* [tiab] OR neoplas* [tiab] OR glioma* [tiab])

AND

(case-control [tiab] OR case-referent [tiab] OR cohort [tiab] OR cross-sectional [tiab] OR comparative [tiab] epidemiol* [tiab] OR “relative risk” [tiab] OR “relative risks” [tiab] OR “odds ratio” [tiab] OR “odds ratios” [tiab] OR “risk ratio” [tiab] OR “risk ratios” [tiab] OR “rate ratio” [tiab] OR “rate ratios” [tiab] OR “prevalence ratio” [tiab] OR “prevalence ratios” [tiab] OR “hazard ratio” [tiab] OR “hazard ratios” [tiab] OR “incidence ratio” [tiab] OR “incidence ratios” [tiab] OR “mortality ratio” [tiab] OR “mortality ratios” [tiab]).

**Fig. 1:**
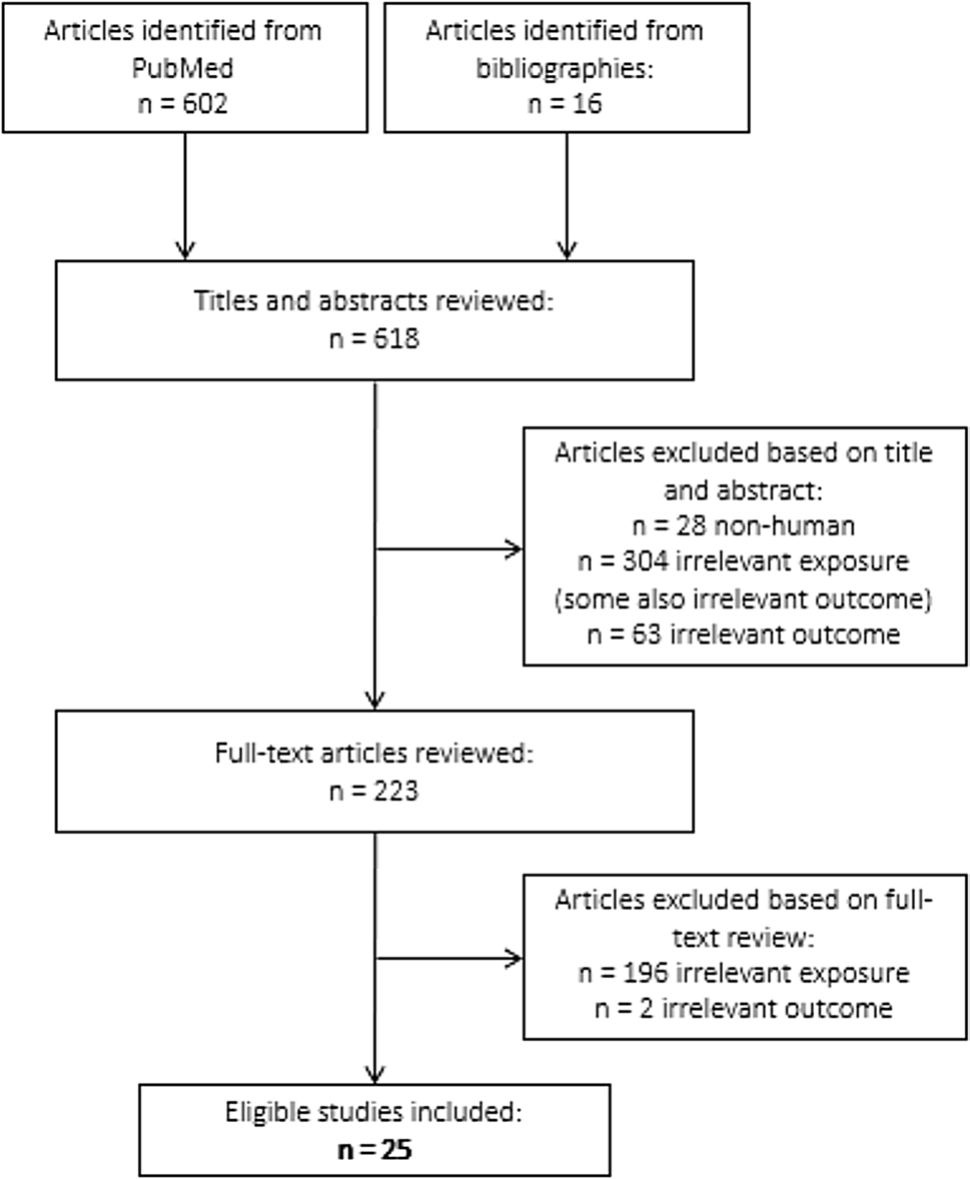
Literature search flow chart

This search string identified 602 results in PubMed as of December 8, 2021. We included 16 additional potentially relevant articles identified from reference lists, bringing the total number of articles considered to 618. After excluding 28 non-human studies, 304 studies of irrelevant exposures (e.g., non-specific categories of pesticides or herbicides, specific pesticides other than glyphosate, occupational titles, or medications), and 63 studies of non-neurological outcomes (e.g., cardiovascular diseases, neoplasms), we considered 223 articles for full-text review. Based on our review of the complete texts of these 223 articles, we excluded 196 studies that did not report original associations with glyphosate exposure and two studies that did not report associations with neurological conditions, leaving 25 articles eligible for inclusion in our systematic review. A flow chart of the literature search process is shown in Fig. 1.

### Data collection

Study characteristics and results were extracted into a spreadsheet with separate fields for first author, publication year, study country, study design, study population (including size, setting, and participation rates), comparison population, exposure assessment method, exposure levels assessed, neurological outcomes assessed, outcome assessment method, confounders adjusted and considered, statistical approach, estimates of association (with interval estimates, e.g., 95% confidence intervals or credible intervals), and funding source. Where applicable, results with multivariable statistical adjustment were prioritized for reporting over unadjusted results, and results for multiple quantitative or semi-quantitative categories of glyphosate exposure were prioritized for reporting over those comparing ever vs. never exposure. Studies were categorized according to whether they assessed neurodegenerative outcomes [e.g., Parkinson disease, amyotrophic lateral sclerosis (ALS)], neurobehavioral outcomes (e.g., depression, cognitive function), neurodevelopmental outcomes (e.g., autism spectrum disorder, neural tube defects), or other or mixed neurological outcomes. Data extraction was conducted by one investigator (ETC) and reviewed for accuracy and completeness by the two other investigators (NUO, JFA).

### Glyphosate exposure assessment

Exposure assessment is critically important in the epidemiology of glyphosate and is, therefore, introduced here in detail as background to inform the exposure assessment quality rating used in our systematic literature review (described in the next section). In general, exposure to pesticides, including glyphosate, is variously defined in epidemiological studies. Here, we refer to “direct exposure metrics” as those that classify exposure based on first-hand or personal application or mixing of a specific pesticide. In contrast, we refer to “indirect exposure metrics” as those that classify exposure to a pesticide based on possible routes other than personal use. For example, some studies classify indirect exposure based on having a household member who mixed or applied a pesticide, or working or living on a farm where a pesticide was applied, without necessarily having applied it oneself. Other studies classify exposure indirectly based on having a residential address within a specified distance from a reported pesticide application. Some studies use an ecological indirect exposure definition, such as total weight of a pesticide applied annually to certain crops within a state or county. The common assumption across all of these exposure metrics is that they provide a valid indication of glyphosate entering the body of persons classified as exposed.

Biomonitoring for glyphosate sheds light on the validity of these various exposure definitions and provides an estimation of the potential range of corresponding doses (namely, the amount of chemical that is biologically internalized). The most comprehensive biomonitoring study for glyphosate is the Farm Family Exposure Study (FFES) (Mandel et al. 2005). The FFES included 48 farmers who applied glyphosate on their farms in Minnesota and South Carolina, along with their immediate family members residing on those farms. Family members aged 4 years and older collected 24-h urine samples for measurement of glyphosate levels on the day before, the day of, and 3 days after the on-study pesticide application. On the day of glyphosate application, 60% of farmers had quantifiable glyphosate in urine [the predominant route of excretion (U.S. EPA 2017b)], whereas 40% of farmers did not; the prevalence of detectable glyphosate declined on post-application days 1–3 (Acquavella et al. 2004). The limit of detection for glyphosate in urine was one part per billion (ppb). The distribution of urinary glyphosate concentrations for applicators was highly skewed, with only a small percentage of values that were appreciably different from the 3-ppb geometric mean. The maximum systemic dose in any farmer was 0.004 mg/kg, and the median systemic dose was 0.0001 mg/kg; methods for calculation of systemic dose, based on amount of glyphosate excreted and adjusting for incomplete excretion, pharmacokinetic recovery, and individual body weight, are described in an appendix to (Acquavella et al. 2005).

These results from the FFES are consistent with those subsequently published (Curwin et al. 2007; Niemann et al. 2015; Solomon 2016). Because all of the farmers personally applied glyphosate, the 40% of farmers with undetectable urinary glyphosate may reasonably be assumed to have had some biological dose of glyphosate below the 1-ppb limit of detection. Accordingly, in the present literature review, we considered personal use of glyphosate to be a valid exposure metric, corresponding to a median systemic dose that is approximately 3–4 orders of magnitude below the level set by various regulatory agencies as an acceptable daily intake [~ 0.3–1 mg/kg/day (EFSA 2015; FAO and WHO 2016; U.S. EPA 2017a)].

Many epidemiological studies of glyphosate employ indirect metrics of exposure, as opposed to direct exposure via personal glyphosate use. For FFES spouses and children who did not take part in the on-study glyphosate application (i.e., excluding those who helped with or were present for glyphosate mixing or application), only two (4%) of 48 spouses and one (2%) of 52 children had detectable glyphosate in their urine on the day of application (Acquavella et al. 2004). For family members without a detectable urinary dose of glyphosate, it is difficult to determine whether any were actually exposed. For instance, some could have been away from the farm at work or at school on the day of glyphosate application, in which case no exposure might have occurred, as evidenced by the lack of a detectable amount of glyphosate in their urine. For indirectly exposed family members in the FFES who had detectable urinary glyphosate values, the maximum systemic doses were 0.00004 mg/kg for spouses and 0.0008 mg/kg for the one child with detectable glyphosate. Thus, an indirect exposure metric is not supported by glyphosate biomonitoring data, which suggest no appreciable dose from indirect exposure scenarios for residents on farms where glyphosate was applied. Accordingly, we determined a priori that in this literature review, we would classify any indirect exposure metrics as being low quality. Although other glyphosate exposure scenarios besides personal occupational use and indirect residential exposure are plausible—for example, spraying at home, ingestion, and dermal contact with recently sprayed crops—no such alternative exposure pathways were assessed in any epidemiological studies of glyphosate and neurotoxicity.

The FFES and other biomonitoring data also cast doubt on the validity of exposure metrics based on residential proximity to a glyphosate application. If measurable glyphosate is extremely rare among family members who were not involved directly in the glyphosate application on their farms, as indicated by these studies (Acquavella et al. 2004; Curwin et al. 2007; Niemann et al. 2015; Solomon 2016), then it is highly unlikely that those residing at some distance from an application would receive any appreciable dose of glyphosate from the attributed application. Reliance on residential proximity to a glyphosate application also introduces uncertainty about whether such individuals were at home and, if so, whether they spent any time outdoors on the day of the proximal application. Accordingly, in our literature review, we determined a priori that all studies with a proximity-based exposure metric would be rated as having low-quality exposure assessment. Similarly, ecological studies do not assign exposures to individuals; hence, there is no way of knowing the likelihood of contact with glyphosate on an individual level. Therefore, we also determined a priori that all ecological studies would be rated as having low-quality exposure assessment because of the obvious limitations of such an approach to exposure classification.

### Risk-of-bias rating

We used the framework developed by the U.S. EPA Office of Pesticide Programs (OPP) to classify each study’s risk of bias in the domains of exposure assessment, outcome assessment, confounder control, statistical approach, and other considerations (e.g., selection bias, other biases that may influence the magnitude of the risk estimate) (U.S. EPA 2016). Each of these five domains was counted once. Ratings were assigned based on consensus among all three investigators.

*Exposure assessment* According to the OPP framework, high quality (i.e., low risk of bias) in exposure assessment calls for use of an exposure metric with an “[a]ccurate and precise relationship with external exposure, internal dose, or target dose, possibly associated with a [mode of action/adverse outcome pathway]” (U.S. EPA 2016). However, none of the epidemiological studies included in this literature review measured glyphosate dose in biological specimens or provided validation of self-reported glyphosate use based on application records or biomonitoring. Therefore, to allow for variation in ratings among the available studies, and consistent with biomonitoring results from the FFES (Acquavella et al. 2004), we considered assessment of self-reported first-hand use of glyphosate, with details on frequency and/or duration of use of glyphosate, to be high quality. We rated assessment of self-reported first-hand use of glyphosate without such details (i.e., ever vs. never use) as being of moderate quality. For the scientific reasons provided in the prior section of this article, any indirect exposure measures other than direct personal use of glyphosate, including assessment based on geographic proximity to glyphosate applications, were rated as low quality.

*Outcome assessment* In line with the OPP framework, we rated outcome assessment as high quality if neurological conditions were classified based entirely on medical records and/or clinical examination by a medical professional. We considered outcome assessment to be moderate quality if it involved a self-reported doctor’s diagnosis of a medical condition, a component of initial self-report followed by validation based on medical records, or ascertainment based on death certificates or diagnosis codes without additional review of medical records. We rated outcome assessment as low quality if it was based entirely on self-report or ecological (population-level) data.

*Confounder control* OPP’s criteria for high-quality confounder control require comprehensively providing “[g]ood control” for “important confounders relevant to [the] scientific question” (U.S. EPA 2016), i.e., potential risk factors for neurological outcomes that may be correlated with glyphosate exposure, as well as standard confounders. Because none of the epidemiological studies identified in this literature review strictly met this definition, we allowed for a range of ratings among the available studies by classifying confounder control as high quality if statistical analyses were adjusted for multiple personal characteristics and other pesticides besides glyphosate; moderate quality if analyses were adjusted for multiple personal characteristics, but no other pesticides besides glyphosate; and low quality if analyses were adjusted for few or no personal characteristics and no other pesticides.

*Statistical approach* Following OPP guidance, we rated the statistical approach as high quality if study authors performed a statistical analysis that was appropriate to the study question and design, supported by adequate sample size, with consideration of approaches to address potential sources of bias. The statistical approach was considered moderate quality if study authors performed an appropriate statistical analysis without consideration of potential bias or with limited sample size; and low quality if statistical comparisons were minimal or not described clearly.

*Other sources of bias* In line with the OPP framework, we considered other potential sources of bias based on a combination of study design and study population, bearing in mind that if properly executed, both cohort and case–control study designs can yield valid results. We rated studies as high quality if they were prospective in design with minimal concerns for selection bias or other sources of bias, for example, based on study recruitment and follow-up methods, inclusion/exclusion criteria, and participation rates; moderate quality if they were prospective in design with moderate concerns for selection bias, or retrospective and population-based in design with high participation; and low quality if they were ecological, cross-sectional, retrospective hospital-based, retrospective population-based with low participation, or case-only in design.

*Overall* Study quality was classified based on the balance of quality ratings across all five domains. Studies that relied on low-quality information on glyphosate exposure are inherently unable to determine whether individuals did or did not receive any dose of glyphosate, rendering them uninformative about possible health impacts. Therefore, we considered all studies with low-quality exposure assessment to be uninformative.

## Results

The methodological characteristics and results of the 13 moderate- and high-quality epidemiological studies of glyphosate and neurological outcomes are summarized in Table 1, grouped by category of neurological outcome. The remaining 12 low-quality studies are summarized in Supplemental Table 1. A heat map of study-specific ratings of the risk of bias in each of the five domains of exposure assessment, outcome assessment, confounder control, statistical approach, and other aspects, as well as the overall study quality rating, is provided in Table 2, along with a brief rationale for each rating. Three studies that used different methods depending on the exposure metric (Beard et al. 2013; Montgomery et al. 2017) or the neurological outcome (Garry et al. 2002) received two ratings in a single domain. A simplified version of the heat map without explanatory text is provided as Supplemental Fig. 1.

**Table 1:** Xxx

Author	Year	Country	Study Design	Study Population	Comparison Subjects	Exposure Assessment	Exposure Levels	Outcome	Outcome Assessment	Confounders Considered	Statistical Approach	Estimate of Association (95% CI or CrI)	Funding Source
*Neurodegenerative outcomes*													
Dhillon et al.	2008	U.S	Hospital-based case–control	100 Parkinson disease patients aged ≥ 50 years recruited from ~ 800 Parkinson disease patients seen at a single neurology practice in East Texas region, excluding patients with multiple sclerosis, schizophrenia, and "Parkinson's plus" diseases; years NR, participation NR (~ 12.5%)	84 controls without Parkinson disease selected from the same neurology practice; participation NR	Self-reported ever personal use/mixing or application of Roundup, Jury, or other glyphosate product and 54 other specific pesticides and pesticide products, as well as various occupations and other occupational exposures, assessed by telephone interviewer, not blinded to case–control status	Ever personally used/mixed or applied Roundup, Jury, or other glyphosate product (54 cases, 43 controls)	Parkinson disease	Parkinson disease diagnosed by a neurologist specializing in movement disorders, using standard clinical/laboratory diagnostic criteria (NR)	None	Chi-square test	Odds ratio = 1.1 (0.6, 2.0)	U.S. Centers for Disease Control and Prevention/National Institute of Occupational Safety and Health
Kamel et al.	2007	U.S	Prospective nested case–control	Agricultural Health Study cohort of 84,738 licensed pesticide applicators (*n* = 52,393; 84% participation) and their spouses (* n* = 32,345; 74% participation), including 22,915 applicators (44% of cohort) who completed a supplemental questionnaire, recruited in 1993–1997 in Iowa and North Carolina, with 5-year follow-up of 57,251 cohort members (68% participation) 83 prevalent Parkinson disease cases (60 applicators, 23 spouses) at enrollment 78 incident Parkinson disease cases (56 applicators, 22 spouses) at follow-up	79,557 controls without Parkinson disease at enrollment, 55,931 controls at follow-up identified within same Agricultural Health Study cohort	Self-reported ever personal mixing or application of glyphosate and 49 other specific pesticides, assessed by written questionnaire at enrollment	Ever use of glyphosate (45 prevalent cases, 46,687 controls; 49 incident cases, 32,686 controls)	Parkinson disease	Prevalent Parkinson disease defined based on self-reported doctor's diagnosis per enrollment questionnaire, applicator questionnaire, or spouse questionnaire, excluding 5073 cohort members with missing data and 25 with conflicting data on enrollment and/or follow-up questionnaires Incident Parkinson disease defined based on self-reported doctor's diagnosis per follow-up telephone interview, excluding prevalent cases and 28,621 cohort members with missing data	Adjusted: age, state, applicator or spouse status, insecticides, herbicides, fungicides, fumigants, organophosphates, organochlorines, carbamates, phenoxyacetates, triazines/triazones	Two-stage hierarchical multivariable logistic regression, with covariates and indicators for specific pesticides with ≥ 4 cases in stage 1, and variables for functional pesticide groups and chemical groups with ≥ 3 pesticides in stage 2	Odds ratio, prevalent disease = 1.0 (0.6, 1.7) Odds ratio, incident disease = 1.1 (0.6, 2.0)	U.S. National Institute of Environmental Health Sciences, U.S. National Cancer Institute
Kamel et al.	2012	U.S.	Prospective nested case–control	Agricultural Health Study cohort of 84,738 licensed pesticide applicators (* n* = 52,393; 84% participation) and their spouses (* n* = 32,345; 74% participation), including ~ 42% of applicators who completed a supplemental questionnaire, recruited in 1993–1997 in Iowa and North Carolina, linked to mortality data through February 7, 2010 41 ALS deaths	84,698 controls without ALS identified within same Agricultural Health Study cohort	Self-reported ever personal mixing or application of glyphosate and 49 other specific pesticides, assessed by written questionnaire at enrollment	Ever use of glyphosate (25 cases, 48,847 controls)	ALS death	Death from ALS as underlying or contributing cause of death identified from death certificates, including 7 deaths (of 41) with medical records available for review by study neurologist using published criteria; 5 diagnosed with ALS (1 definite, 2 probable, 2 possible), 1 with progressive bulbar palsy, 1 indeterminate	Adjusted: age, sex Considered: smoking, education, state, head injury	Multivariable logistic regression	Odds ratio = 1.2 (0.6, 2.5)	U.S. National Institute of Environmental Health Sciences, U.S. National Cancer Institute
Montgomery et al.	2017	U.S	Prospective nested case–control	Agricultural Health Study cohort of 84,739 licensed pesticide applicators (* n* = 52,394; 84% participation) and their spouses (* n* = 32,345; 74% participation), including a supplemental questionnaire completed by 53% of controls and 72% of cases, recruited in 1993–1997 in Iowa and North Carolina, with follow-up interviews in 1993–2003 and 2005–2010 (68% participation), restricted to 41,863 cohort members aged < 50 y on September 1, 2007, without retinal or macular degeneration at enrollment 161 incident AMD cases	39,108 controls without AMD or possible AMD identified within same Agricultural Health Study cohort	Self-reported ever personal mixing or application of glyphosate and 49 other specific pesticides, including duration in years and frequency in days per year, assessed by written questionnaire at enrollment	Ever use of glyphosate (103 cases, 23,493 controls), including cumulative days of use among applicators: 0: 15 cases, 5104 controls > 0–10: 18 cases, 4929 controls > 10–100: 33 cases, 7403 controls > 100: 28 cases, 4783 controls	AMD	Validated self-reported doctor's diagnosis of AMD at either follow-up telephone interview, with self-report affirmed by screening call and confirmed by treating physician with supporting pathology or by study ophthalmologist based on retinal photographs, excluding self-reported prevalent doctor's diagnosis of retinal or macular degeneration per enrollment questionnaire (* n* = 324); further classified as early stage, late-stage, or unknown-stage	Adjusted: age, sex, smoking at enrollment; in some models: strongly correlated pesticide Considered: body mass index, education, state, sun exposure hours per day	Multivariable logistic regression	Odds ratio, ever use = 1.4 (0.99, 2.0) Odds ratio, ever use, me* n* = 1.8 (1.0, 3.1) Odds ratio, ever use, wome* n* = 1.2 (0.7, 2.0) Odds ratio, ever use, early AMD = 1.5 (0.8, 2.7) Odds ratio, ever use, late AMD = 1.3 (0.8, 2.3) Odds ratio, ever use, late vs. early AMD = 0.9 (0.4, 2.0) Odds ratio, ever use, adjusted for malathio* n* = 1.1 (not significant) Odds ratio, ever use, adjusted for carbaryl = 1.3 (not significant) Odds ratio, ever use, adjusted for 2,4-D = 1.3 (not significant) Odds ratio, > 0–10 days vs. 0 = 1.3 (0.6, 2.5) Odds ratio, > 10–100 days vs. 0 = 1.7 (0.9, 3.1) **Odds ratio, > 100 days vs. 0 = 2.6 (1.4, 4.9)** **p-trend = 0.002**	U.S. National Institute of Environmental Health Sciences, U.S. National Cancer Institute
Shrestha et al.	2020	U.S	Prospective cohort	Agricultural Health Study cohort of 38,274 male licensed pesticide applicators (84% initial participation; 44% participation in supplemental questionnaire) and 27,836 spouses (75% initial participation) recruited in 1993–1997 in Iowa and North Carolina, with completion of at least one follow-up interview in 1993–2003, 2005–2010, or 2013–2016 or a Parkinson disease validation screening questionnaire 491 incident Parkinson disease cases (373 applicators, 118 spouses), 65,619 non-cases 106 prevalent Parkinson disease cases	Cohort members without glyphosate exposure	Self-reported ever personal mixing or application of glyphosate and 49 other specific pesticides, including duration in years and frequency in days per year, assessed by written questionnaire at enrollment; in phase 2 (2–10 y after enrollment, mean 5 y), self-reported days of use of glyphosate in year prior to interview or in most recent year assessed by telephone interview; intensity-weighted lifetime days of use among pesticide applicators calculated as years of use × days per year × exposure intensity, with weights based on mixing practices, application methods, repair status, and personal protective equipment use	Ever use of glyphosate (291 cases, 35,406 non-cases) Through enrollment: Never use of glyphosate, applicators only (86 cases, 8,307 non-cases) > 0– ≤ 677 intensity-weighted lifetime days of glyphosate use (106 cases, 8,996 non-cases) > 677– ≤ 2,604 intensity-weighted lifetime days of glyphosate use (91 cases, 9,313 non-cases) > 2,604 intensity-weighted lifetime days of glyphosate use (73 cases, 9,015 non-cases) Through phase 2: Never use of glyphosate, applicators only (62 cases, 5247 non-cases) > 0– ≤ 970 intensity-weighted lifetime days of glyphosate use (132 cases, 9965 non-cases) > 970–≤ 3352 intensity-weighted lifetime days of glyphosate use (84 cases, 10,318 non-cases) > 3352 intensity-weighted lifetime days of glyphosate use (77 cases, 10,018 non-cases)	Parkinson disease	Incident Parkinson disease defined based on self-reported doctor's diagnosis per follow-up interview or linkage to National Death Index and state death registries; self-reported cases through phase 2 previously confirmed by movement disorder specialists via structured clinical examinations and medical records (confirmed in 84% of self-reported cases); all incident and prevalent self-reported cases through phase 4 re-contacted for validation through self- or proxy completion of detailed screening questionnaire on Parkinson disease diagnosis, symptoms, characteristics, and treatment, with adjudication by a movement disorder specialist, and review of medical records if consented (* n* = 65; 91% confirmed, 9% questionable); excluded prevalent cases and those without information on age at diagnosis, without supporting symptoms or medications, or with inconsistent survey responses	Adjusted: applicator status, sex, state, smoking, alcohol, education, top four pesticides with Spearman correlation coefficient of ≥ 0.40	Multivariable Cox proportional hazards regression with attained age as time scale, left-truncated at enrollment, and stratification of baseline hazard by median age (63 y) when proportional hazards assumption failed; sensitivity analysis using inverse probability of censoring weights to adjust for loss to follow-up	Hazard ratio, ever use, applicators and spouses = 1.10 (0.87, 1.39) Hazard ratio, ever use, applicators = 1.02 (0.79, 1.30) Hazard ratio, ever use, spouses = 1.44 (0.92, 2.25) Hazard ratio, > 0– ≤ 677 intensity-weighted lifetime days through enrollment, applicators = 1.17 (0.88, 1.55) Hazard ratio, > 677– ≤ 2,604 days = 0.99 (0.73, 1.33) Hazard ratio, > 2,604 days = 0.85 (0.62, 1.17) p-trend = 0.09 Hazard ratio, > 0– ≤ 970 intensity-weighted lifetime days through phase 2, applicators = 1.21 (0.88, 1.65) Hazard ratio, > 970– ≤ 3,352 days = 0.92 (0.64, 1.34) Hazard ratio, > 3352 days = 0.88 (0.62, 1.25) p-trend = 0.10 No significant associations after stratification by ≤ 10 y vs. > 10 y follow-up, inclusion of prevalent cases, use of inverse probability weights, or classification of ever use through phase 2	U.S. National Institute of Environmental Health Sciences, U.S. National Cancer Institute
*Neurobehavioral outcomes*													
Beard et al.	2013	U.S	Prospective cohort	Agricultural Health Study cohort of 16,893 wives of licensed pesticide applicators who completed a take-home questionnaire (75% participation), recruited in 1993–1997 in Iowa and North Carolina, with 5-year follow-up (62% participation), excluding depression at enrollment 1,054 incident depression cases and 15,839 non-cases at follow-up	Cohort members without glyphosate exposure	Self-reported ever personal mixing or application of glyphosate (direct) or ever spousal mixing or application of glyphosate (indirect) if never personally used pesticides, including 49 other specific pesticides and 11 pesticide classes, assessed by written questionnaire at enrollment	Ever personal use of glyphosate (359 cases, 6,017 total) Husbands' ever use of glyphosate (if never personally used pesticides; 330 cases, 4935 total)	Depression	Self-reported doctor's diagnosis of depression per follow-up telephone interview, excluding self-reported prevalent doctor's diagnosis of depression per enrollment questionnaire (* n* = 2252) and those with missing data or self-reported age at diagnosis before enrollment on follow-up interview	Adjusted: age, diabetes, education, state Considered: race/ethnicity, number of children in family, farm size, alcohol in past year, smoking, number of doctor visits in past year, heart disease, number of years lived or worked on a farm, job held off of farm, solvent (other than gasoline) exposure at longest held non-farm job, ever use of any pesticides, husbands' age, husbands' depression status, husbands' use of individual pesticides, most strongly correlated pesticide	Multivariable log-binomial regression, with various regression models to calculate stabilized weights accounting for confounding and selection bias from loss to follow-up, multiplied to obtain overall stabilized weights used for inverse probability weighting; sensitivity analysis using Cox proportional hazards regression with estimated date of depression diagnosis	Risk ratio, personal use = 0.95 (0.83, 1.08) Risk ratio, spousal use = 1.04 (0.84, 1.30)	U.S. National Institute of Environmental Health Sciences, U.S. National Cancer Institute
Beard et al.	2014	U.S	Prospective nested case–control	Agricultural Health Study cohort of 21,208 male licensed pesticide applicators (40% of cohort with 84% initial participation), including a supplemental questionnaire completed by 56% of analytic cohort, recruited in 1993–1997 in Iowa and North Carolina, with 5-year follow-up (68% participation) 1702 depression cases, including 474 at enrollment but not follow-up, 540 at enrollment and follow-up, and 688 at follow-up but not enrollment	19,506 controls without depression at enrollment and follow-up identified within same Agricultural Health Study cohort	Self-reported ever personal mixing or application of glyphosate and 49 other specific pesticides and 10 pesticide classes	Ever personal use of glyphosate (376 pre-enrollment only cases, 426 pre-enrollment and follow-up cases, 540 follow-up only cases, 15,053 controls)	Depression	Self-reported doctor's diagnosis of depression per enrollment questionnaire (including self-reported doctor's diagnosis of depression requiring medication or shock therapy per supplemental questionnaire) and/or follow-up telephone interview, excluding 1894 cohort members with missing depression data	Adjusted: age, diabetes, education, state Considered: marital status, number of children in family, alcohol in past year, smoking, farm size, use of chemical-resistant gloves when handling pesticides, number of doctor visits in past year, number of years lived or worked on a farm, job held off of farm, solvent (other than gasoline) exposure in longest-held non-farm job, most strongly correlated pesticide	Multivariable polytomous logistic regression, with various regression models to calculate stabilized weights accounting for confounding, missing covariate data, missing supplemental questionnaire (if applicable), and selection bias from loss to follow-up, multiplied to obtain overall stabilized weights used for inverse probability weighting	Odds ratio, pre-enrollment depression only = 1.2 (0.9, 1.6) Odds ratio, pre-enrollment and follow-up depressio* n* = 1.1 (0.9, 1.4) Odds ratio, follow-up depression only = 1.1 (0.9, 1.3) *p* = 0.80 for difference among odds ratios	U.S. National Institute of Environmental Health Sciences, U.S. National Cancer Institute, U.S. National Institute for Occupational Safety and Health
Fuhrimann et al.	2021	Uganda	Cross-sectional	288 smallholder farmers aged ≥ 18 years, including approximately equal numbers of conventional farmers sampled from lists provided by local leaders, and organic farmers sampled from a list provided from a local non-governmental organization, using snowball recruiting (participation NR), Wakiso District, Uganda, 2017	Subjects without glyphosate exposure	Self-reported use of glyphosate and 13 other specific pesticides in 12 months before study; exposure intensity score calculated as (mixing + application) × overall average personal protective equipment use × time interval between pesticide application and change of clothes × time interval between application and shower; also multiplied by yearly number of application days	Glyphosate application (208 subjects) Glyphosate mixing (191 subjects) Glyphosate exposure intensity score (media* n* = 6.1, interquartile range = 3.0) Glyphosate yearly application days (media* n* = 9, interquartile range = 26) Glyphosate yearly exposure-intensity-score-weighted days (media* n* = 51.5, interquartile range = 179.5)	Language, memory, attention, executive function, and motor function	Neurobehavioral tests administered by trained psychometrician, including Semantic Verbal Fluency, Phonetic Verbal Fluency (language); Color Trail Part 2 (inhibition/flexibility); Digit Span backward (working memory); Trail Making Test A (processing speed); Digit Symbol Substitution Test, Digit Vigilance (sustained attention); Benton Visual Retention Test (recognition memory); Digit Span forward (short-term memory); Purdue pegboard (perceptual motor, fine motor ability, coordination); Finger Tapping test (hand motor speed)	Adjusted: pesticide applicator status, age, education, psychometrician, language of assessment, sex, literacy, alcohol use, history of head injury, HIV status	Bayesian model averaging to compute inclusion probability for each predictor by summing posterior model probabilities over models including that predictor, with Jeffreys‐Zellner‐Siow prior for regression coefficients and beta‐binomial prior for model space, accounting for multiple testing across different models	**Benton Visual Retention: marginal inclusion probability = 0.665, slope per interquartile increase in exposure intensity score = -0.103 (-0.236, 0)** Finger Tapping, dominant hand: marginal inclusion probability = 0.483, slope = -0.217 (-0.712, 0) Trail Making A, log10: marginal inclusion probability = 0.176, slope = 0.002 (0, 0.013) Finger Tapping, non-dominant hand: marginal inclusion probability = 0.235, slope = -0.087 (0.531, 1e-04) Digit Symbol: marginal inclusion probability = 0.176, slope = -0.068 (-0.522, 0) Semantic Verbal Fluency: marginal inclusion probability = 0.078, slope = 0.007 (0, 0.09) Other neurobehavioral measures: marginal inclusion probability of empty model ≥ 0.5	Swiss Network for International Studies, Swiss National Science Foundation
**Neurodevelopmental outcomes**													
Juntarawijit et al.	2020	Thailand	Hospital-based case–control	442 children aged < 5 years with suspected developmental delay identified from National Child Developmental Screening Program in 15 of 21 randomly selected hospitals in one rural area (Bang Rakam district) and 10 of 30 randomly selected hospitals in one urban area (Muang district), Phitsanulok Province, Thailand, 2019; 87% participation	413 controls with normal development identified from National Child Developmental Screening Program at same hospital, matched on age, sex, area of residence; 81% participation	Maternal self-reported ever prenatal or postnatal use of glyphosate and 13 other pesticides, reported in questionnaire administered by trained village health volunteers	Ever maternal use of glyphosate (33 cases, 34 controls) Ever prenatal maternal use of glyphosate (29 cases, 32 controls) Ever postnatal maternal use of glyphosate (16 cases, 11 controls)	Suspected developmental delay	Suspected developmental delay identified based on failure of one or more skills (gross motor, fine motor, receptive language, expressive language, and personal and social) assessed at ages 9, 18, 30, and 42 months using Developmental and Surveillance Promotion Manual, modified from Denver Development Screening Test II, conducted by trained nurse or health personnel as part of National Child Developmental Screening Program	Adjusted: maternal age at pregnancy, education, occupation, income, chronic disease, alcohol consumption, gestational age, birth order, delivery method, birth weight, and breastfeeding	Multivariable logistic regression	Odds ratio, glyphosate ever, adjusted = 0.93 (0.46, 1.90) Odds ratio, glyphosate ever, unadjusted = 0.90 (0.54, 1.47) Odds ratio, glyphosate prenatal, adjusted = 0.92 (0.45, 1.91) Odds ratio, glyphosate prenatal, unadjusted = 0.83 (0.49, 1.40) Odds ratio, glyphosate postnatal, adjusted = 1.32 (0.49, 3.55) Odds ratio, glyphosate postnatal, unadjusted = 1.37 (0.63, 2.98)	Faculty of Nursing, Naresuan University, Thailand
*Other neurological outcomes*													
Fuhrimann et al.	2022	Uganda	Cross-sectional	253 smallholder farmers aged ≥ 18 years, including approximately equal numbers of conventional farmers chosen using random clustered convenience sampling from lists provided by local leaders, and organic farmers sampled from a list provided from a local non-governmental organization in Wakiso District, Uganda, using snowball recruiting in 2017 (participation NR), with follow-up interview of initial study subjects in 2019 (84% follow-up participation)	Subjects without glyphosate exposure	Self-reported use of glyphosate and 29 other specific pesticides on crops, livestock, or household in past year or 7 days prior to interview	Glyphosate use in past week (31 subjects) Glyphosate use in past year but more than 1 week ago (120 subjects)	Sleep problems	Self-reported sleep problems during past 1 week assessed by modified 12-item Medical Outcomes Study Sleep Scale questionnaire, used to derive measures of overall sleep problems (6 items or 9 items), sleep disturbance (4 items), sleep inadequacy (2 items), daytime somnolence (3 items), snoring (1 item), awakening short of breath or with a headache (1 item), and non-optimal sleep quantity (1 item); proportionally transformed to 100-point scale and dichotomized at 30 points. Modified from standard sleep scale by changing usual 1-month measurement period to 1 week, and changing 6-point Likert scale from "not at all" to "all the time" to 8-point Likert scale from 0 to 7 days	Adjusted: age, sex, current alcohol consumption, body mass index, sleep disruption during past week (yes or no) by mosquitoes, bedbugs, noise, infectious disease, wearing actimeter, or any other reason	Multivariable logistic regression	Odds ratio for 6-item sleep problem index, glyphosate use in past year but more than 1 week ago = 1.29 (0.64, 2.59) **Odds ratio for 6-item sleep problem index, glyphosate use in past week = 3.75 (1.24, 11.8)** **6-item sleep problem index and frequency of glyphosate use in past week (0, 1–2, or > 2 days): odds ratio NR, *** p* **< 0.05** Results NR for 9-item sleep problem index or any of 6 sleep dimensions measured; not significantly associated with frequency of glyphosate use in past week (0, 1–2, or > 2 days)	Swiss National Science Foundation; Swiss Network for International Studies; CropLife Europe
Shrestha et al.	2018	U.S	Prospective nested case–control	Agricultural Health Study cohort of 20,591 male licensed farmers, recruited in 1993–1997 in Iowa and North Carolina (84% participation at enrollment), with follow-up interview in 2013–2015 (46% participation) 1623 dream-enacting behavior cases	16,441 controls without dream-enacting behaviors identified within same Agricultural Health Study cohort	Self-reported ever personal mixing or application of glyphosate and 49 other specific pesticides	Ever personal use of glyphosate (1143 cases)	Dream-enacting behaviors	Self-reported dream-enacting behaviors based on question "Have you ever been told, or suspected yourself, that you seem to 'act out dream' while sleeping? For example, punching or flailing arms in the air, shouting, or screaming while asleep." If yes, further classified by frequency of symptoms (< 3 times in life, < 1/month, 1–3 month, 1/week, or > 1/week), with sensitivity analyses excluding 179 subjects diagnosed with Parkinson disease or restricting to cases with ≥ 3 lifetime episodes	Adjusted: age, smoking, alcohol, marital status, education, state, head injury; in some models: other pesticides and functional/chemical classes of pesticides with statistically significant associations before mutual adjustment	Multivariable logistic regression, with logistic regression to calculate stabilized weights accounting separately for loss of participants and for missing covariates, multiplied to obtain overall stabilized weights used for inverse probability weighting	**Odds ratio, unadjusted for other pesticides = 1.3 (1.1, 1.5)** **Odds ratio, unadjusted, excluding Parkinson disease = 1.3 (1.1, 1.6)** **Odds ratio, unadjusted, ≥ 3 lifetime episodes = 1.3 (1.1., 1.5)** **Odds ratio, adjusted for other pesticides = 1.2 (1.0, 1.4)** **Odds ratio, adjusted, excluding Parkinson disease = 1.2 (1.0, 1.5)** Odds ratio, adjusted, ≥ 3 lifetime episodes = 1.2 (1.0, 1.4)	U.S. National Institute of Environmental Health Sciences, U.S. National Cancer Institute, Michigan State University
Shrestha et al.	2021	U.S	Prospective nested case–control	Agricultural Health Study cohort of 20,409 licensed pesticide applicators recruited in 1993–1997 in Iowa and North Carolina (84% participation at enrollment, 44% with supplemental questionnaire), with completed follow-up interview in 2013–2016 (participatio* n* ~ 40%) 2,069 cases of olfactory impairment	18,340 controls without olfactory impairment identified within same Agricultural Health Study cohort	Self-reported ever personal mixing or application of glyphosate and 49 other specific pesticides, including duration in years and frequency in days per year, assessed by written questionnaire at enrollment; in phase 2, self-reported days of use of glyphosate in year prior to interview or in most recent year assessed by telephone interview; intensity-weighted lifetime days of use among pesticide applicators at enrollment or through phase 2 calculated as years of use × days per year × exposure intensity, with weights based on mixing practices, application methods, repair status, and personal protective equipment use	Ever personal use of glyphosate (1,678 cases with no time restriction, 991 cases ≤ 10 y before phase 4 [third follow-up], 14,086 controls) Never use of glyphosate (381 unrestricted cases, 226 cases ≤ 10 y before phase 4, 4,163 controls) > 0–672 intensity-weighted lifetime days of glyphosate use (573 unrestricted cases, 340 cases ≤ 10 y before phase 4, 4590 controls) > 672–2610 intensity-weighted lifetime days of glyphosate use (523 unrestricted cases, 302 cases ≤ 10 y before phase 4, 4748 controls) > 2610 intensity-weighted lifetime days of glyphosate use (567 unrestricted cases, 342 cases ≤ 10 y before phase 4, 4572 controls)	Olfactory impairment	Self-reported olfactory impairment based on question "Do you suffer from a loss of sense of smell or significantly decreased sense of smell?" If yes, further classified by timing of when loss of sense of smell began (≤ 1, 1–5, 5–10, or > 10 y before phase 4 interview [third follow-up])	Adjusted: age, sex, state, education, smoking, other farming tasks, correlated pesticides with Spearman ρ ≥ 0.40	Multivariable logistic regression; sensitivity analyses using inverse probability of censoring weights to adjust for loss to follow-up	**Odds ratio, ever use = 1.33 (1.18, 1.50)** **Odds ratio, ever use, onset ≤ 10 y prior = 1.31 (1.13, 1.53)** **Odds ratio, > 0–672 intensity-weighted lifetime days = 1.38 (1.21, 1.59)** **Odds ratio, > 672–2,610 days = 1.22 (1.06, 1.41)** **Odds ratio, > 2,610 days = 1.41 (1.22, 1.62)** **p-trend < 0.01** **Odds ratio, > 0–672 intensity-weighted lifetime days, onset ≤ 10 y prior = 1.39 (1.16, 1.65)** Odds ratio, > 672–2610 days, onset ≤ 10 y prior = 1.17 (0.98, 1.41) **Odds ratio, > 2610 days, onset ≤ 10 y prior = 1.40 (1.17, 1.68)** **p-trend = 0.02** No appreciable changes in sensitivity analyses excluding those with positive or unknown history of head injury, excluding those self-reporting Parkinson disease, using unweighted lifetime days of use, using average days/year of use, using intensity-weighted lifetime days through phase 2, or using inverse probability of censoring weights	U.S. National Institute of Environmental Health Sciences, U.S. National Cancer Institute, Michigan State University, Parkinson's Foundation, Office of the Assistant Secretary of Defense for Health Affairs through the Parkinson's Research Program
Zhang et al.	2018	China	Prospective cohort	218 farmers identified as main pesticide users in 20–25 farm households randomly selected from two villages per county, two counties per province in Guangdong, Jiangxi, and Hebei Provinces, China, followed from beginning to end of 2012 growing season; 89% participation	Cohort members without glyphosate exposure	Self-reported amount of glyphosate and other specific agricultural pesticides used in 2012, including chemical name, active ingredient percentage, amount used in kg, and date and duration of spray, recorded after each spray application; pesticide application records checked every other week, pesticide containers saved and checked twice per month	Glyphosate applied per farmer in 2012: mea* n* = 0.62 kg (52% of total herbicides applied)	Peripheral nerve conduction velocity	Conventional peripheral nerve conduction studies implemented at beginning of planting season (March 2012) and prior to but close to end of crop harvest (March 2012 in Jiangxi and Hebei; December 2012 in Guangdong), including 22 parameters of peripheral nerve conduction examined using surface electrodes with standard placement; classified as nerve conduction velocity, motor conduction velocity, sensory conduction velocity, distal motor latency, amplitude of action potential, amplitude of compound muscle action potential, and amplitude of sensory nerve action potential, categorized as normal vs. abnormal or aggregated into counts of abnormal parameters	Adjusted: age, sex, smoking, alcohol consumption, use of personal protective measures (e.g., wearing masks, gloves, or clothes with long sleeves), diabetes mellitus, body mass index, baseline peripheral nerve conduction, and other classes of pesticides (non-glyphosate herbicides or organophosphorus, organonitrogen, organosulfur, pyrethroid, and other insecticides and fungicides)	Multivariable logistic and negative binomial regression	Odds ratio for abnormal overall nerve conduction velocity = 0.70 (0.38, 1.30) Incidence rate ratio for # abnormal parameters of overall nerve conduction velocity = 0.86 (0.67, 1.10) Odds ratio for abnormal motor nerve conduction velocity = 1.34 (0.30, 6.03) Incidence rate ratio for # abnormal parameters of motor nerve conduction velocity = 1.11 (0.81, 1.53) Odds ratio for abnormal sensory nerve conduction velocity = 0.64 (0.35, 1.18) Incidence rate ratio for # abnormal parameters of sensory nerve conduction velocity = 0.74 (0.52, 1.06) Odds ratio for abnormal distal motor latency = 1.05 (0.81, 1.37) Incidence rate ratio for # abnormal parameters of distal motor latency = 1.02 (0.85, 1.22) Odds ratio for abnormal overall amplitude = 1.21 (0.75, 1.97) Incidence rate ratio for # abnormal parameters of overall amplitude = 0.96 (0.65, 1.43) Incidence rate ratio for # abnormal parameters of motor amplitude = 1.25 (0.67, 2.34) Incidence rate ratio for # abnormal parameters of sensory amplitude = 1.04 (0.49, 2.19)	National Natural Science Foundation of China

Statistically significant associations are shown in bold font

*ALS* amyotrophic lateral sclerosis, *AMD* age-related macular degeneration, *CI* confidence interval, *CrI* credible interval, *NR* not reported

**Table 2:**
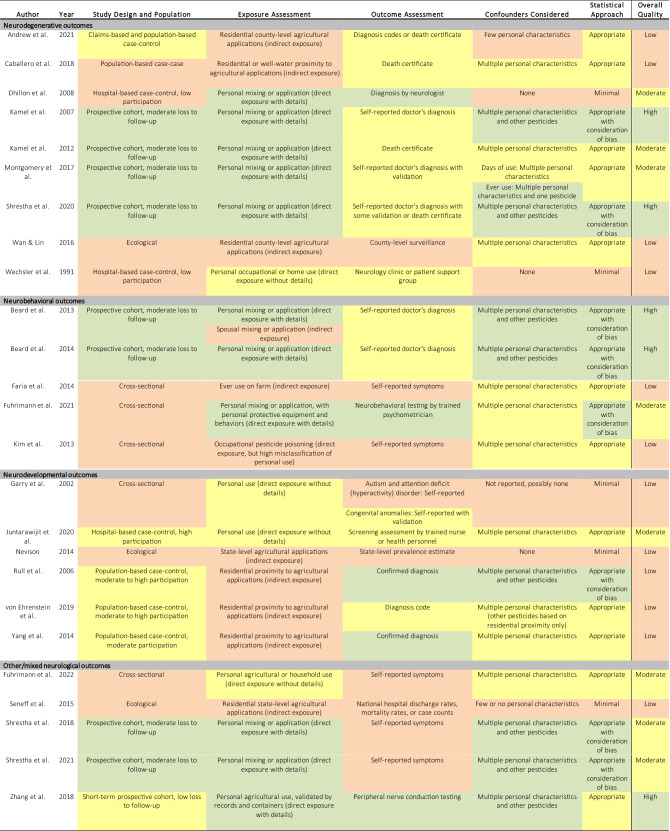
Xxx

### Neurodegenerative outcomes

We rated two studies of neurogenerative outcomes as high quality overall (Tables 1, 2). These were overlapping analyses of Parkinson disease in the Agricultural Health Study (AHS) cohort: the first study evaluated prevalent and incident cases in the full initial cohort (Kamel et al. 2007), and the second study was an updated analysis including only incident cases, based on cohort members with at least one follow-up assessment (Shrestha et al. 2020). The AHS is a prospective cohort study of more than 57,000 licensed pesticide applicators (mostly farmers) from Iowa and North Carolina and more than 32,000 of their spouses, all of whom were initially enrolled in 1993–1997 by completing questionnaires about agricultural practices, including personal mixing or application of 50 specific pesticides, as well as lifestyle and health factors (Agricultural Health Study 2021). Pesticide applicators reported additional information on frequency and duration of pesticide use, as well as mixing practices, application methods, repairing of application equipment, and use of personal protective equipment. (Most studies of neurological outcomes in the AHS, however, provided results only for ever vs. never glyphosate use.) Follow-up interviews or questionnaires were completed in 1999–2003, 2005–2010, and 2013–2015, and cohort members were linked to vital statistics and state cancer registries to ascertain cause-specific mortality and cancer incidence. Certain self-reported health outcomes were confirmed by medical records or clinical examination.

As summarized in Tables 1 and 2, although both of the AHS studies of Parkinson disease relied to some extent on self-reported health endpoints, resulting in a moderate-quality rating for outcome assessment, they were considered to have relatively high-quality (i.e., low-bias) exposure assessment, confounder control, statistical approach, and other aspects of study design and study population. Both high-quality studies of neurodegenerative outcomes estimated near-null associations (i.e., relative risks close to 1.0) between glyphosate use and risk of Parkinson disease (Kamel et al. 2007; Shrestha et al. 2020) (Table 1).

Two other AHS cohort studies of neurodegenerative outcomes, ALS mortality and age-related macular degeneration, were rated as moderate quality overall (Kamel et al. 2012; Montgomery et al. 2017), as was a hospital-based case–control study of Parkinson disease in East Texas (Dhillon et al. 2008) (Tables 1, 2). These AHS studies were downgraded to moderate quality based on their limited adjustment for confounding, combined with ascertainment of ALS based primarily on death certificates (Kamel et al. 2012), which are prone to classification error (Chio et al. 1992; Middleton et al. 2011); or ascertainment of age-related macular degeneration based initially on self-report, followed by medical confirmation of only positive reports (Montgomery et al. 2017). Risk of age-related macular generation was positively associated with the highest category of glyphosate exposure (> 100 lifetime days), but ever use of glyphosate was not associated with risk after more rigorous confounder adjustment (Montgomery et al. 2017). No association was observed between glyphosate use and death from ALS (Kamel et al. 2012).

The hospital-based case–control study of Parkinson disease in East Texas, also rated as moderate quality, found no association between ever use of glyphosate and risk of neurologist-diagnosed Parkinson disease (Dhillon et al. 2008) (Tables 1, 2). This study design had substantial problems with selection bias and confounding, but had high-quality exposure and outcome assessment.

The remaining four studies of neurodegenerative outcomes were rated as low quality overall (Table 2 and Supplemental Table 1). These included three ecological or semi-ecological studies of ALS or Parkinson disease that used regional data on agricultural applications of glyphosate (Andrew et al. 2021; Caballero et al. 2018; Wan and Lin 2016); and a small, pilot hospital-based case–control study of Parkinson disease that was rated as low quality due to its inclusion of some unvalidated cases identified from patient support groups, as well as its absence of any adjustment for confounding (Wechsler et al. 1991). Due to their serious methodological weaknesses, which limited their ability to provide scientific insight on any potential neurotoxic effects of glyphosate, these low-quality studies were not considered further in our weight-of-evidence evaluation.

### Neurobehavioral outcomes

Two studies of neurobehavioral outcomes were rated as high quality overall; these were non-overlapping analyses of depression (one in women and one in men) in the prospective AHS cohort (Beard et al. 2013; Beard et al. 2014) (Tables 1, 2). Both of these studies reported near-null results, with relative risks close to 1.0, for associations between glyphosate use and risk of depression (Beard et al. 2013; Beard et al. 2014).

In a moderate-quality cross-sectional study of a convenience sample of 288 Ugandan farmers, self-reported glyphosate use, which was classified semi-quantitatively using detailed exposure information, was associated with poorer visual retention, but not with several other neurobehavioral outcomes assessed using a standard battery of tests (Fuhrimann et al. 2021) (Tables 1, 2).

The other two studies of neurobehavioral outcomes were considered uninformative for our weight-of-evidence evaluation due to their poor methodological quality (Table 2 and Supplemental Table 1). These were cross-sectional analyses with low-quality exposure assessment based on farm-level but not personal glyphosate use in relation to self-reported minor psychiatric disorders (Faria et al. 2014); and self-reported acute occupational glyphosate poisoning in relation to self-reported depressive symptoms (Kim et al. 2013). In the latter study (Kim et al. 2013), farmers were classified as exposed (i.e., poisoned) if they retrospectively reported having experienced any of 21 non-specific symptoms and signs (nausea, vomiting, diarrhea, sore throat, runny nose, dyspnea, headache, dizziness, hyperactivity, profuse sweating, blurred vision, paresthesia, slurred speech, paralysis, chest pain, syncope, muscle weakness, skin irritation, eye irritation, lacrimation, and fatigue) within 48 h of using pesticides in the prior year. Subjects who did not report such episodes were classified as non-poisoned; however, no distinction was made between non-poisoned farmers who did and did not apply glyphosate. Given the non-specific nature of the exposure assessment and the inability to distinguish between glyphosate use and non-use, we rated the exposure metric in this study as low quality.

### Neurodevelopmental outcomes

None of the epidemiological studies of glyphosate exposure and neurodevelopmental outcomes were rated as high quality (Table 2). Among the six identified studies of neurodevelopmental endpoints, only one—a hospital-based case–control study of suspected developmental delay among young children in Thailand (Juntarawijit et al. 2020)—merited a rating of moderate quality. This study found no association between maternal self-reported prenatal, postnatal, or overall use of glyphosate and suspected developmental delay among children under age 5 years who participated in Thailand’s National Child Developmental Screening Program (Juntarawijit et al. 2020) (Tables 1, 2).

Notably, five of the six studies of neurodevelopmental outcomes were rated as low quality (Table 2 and Supplemental Table 1). These included one ecological study (ranked as lowest in quality) that was considered low quality due to its use of state-level estimates of glyphosate agricultural applications and autism prevalence over time (Nevison 2014); three semi-ecologic studies that were considered low quality due to their use of residential proximity to glyphosate applications as the exposure metric in relation to neural tube defects, autism spectrum disorder, and neural tube defects and orofacial clefts, respectively (Rull et al. 2006; von Ehrenstein et al. 2019; Yang et al. 2014); and a cross-sectional analysis that was rated as low quality due to its reliance on unvalidated parent-reported data on childhood autism or attention deficit/hyperactivity disorder (as well as confirmed parent-reported data on congenital central nervous system anomalies), cross-sectional design, and no or minimal confounder adjustment (Garry et al. 2002). Due to their low overall quality, these studies were not considered further in our weight-of-evidence evaluation.

### Other and mixed neurological outcomes

One high-quality short-term prospective cohort study evaluated glyphosate exposure in relation to peripheral nerve conduction (categorized as an “other” neurological outcome) among Chinese farmers, with relatively high-quality exposure assessment, outcome assessment, confounder control, and statistical approach (Zhang et al. 2018) (Tables 1, 2). In this study, 218 farmers in three provinces in China were followed for the 2012 planting season. The farmers kept detailed records of pesticide use throughout the duration of the study, and nerve conduction parameters were measured at the beginning and near the end of the planting season. Strengths of this study include the use of well-controlled nerve conduction tests and careful real-time assessment of pesticide use, including biweekly checking of farmers’ records and pesticide containers. Multivariable regression was used to control for several potential confounding factors, including other pesticide classes. Limitations are the short 1-year timeframe considered for potential chronic effects of pesticides, the lack of measurement of biological dose of glyphosate, and not having more extended pesticide histories for the subjects. Use of glyphosate was not found to be associated with any measure of nerve conduction, whether classified as abnormal vs. normal or based on the total number of abnormal parameters noted in a given domain.

Two AHS analyses were rated as moderate quality due to their reliance on exclusively self-reported, unvalidated neurological outcomes, namely, dream-enacting behaviors and olfactory impairment (Shrestha et al. 2018; Shrestha et al. 2021) (Tables 1, 2). Both of these studies reported mostly positive associations with glyphosate use, including semi-quantitative measures of intensity-weighted cumulative glyphosate use in the analysis of olfactory impairment (Shrestha et al. 2021).

A cross-sectional analysis in the Ugandan farmer population, also rated as moderate quality, showed that self-reported glyphosate use in the past week, but not otherwise in the past year, was associated with a higher prevalence of self-reported overall sleep problems in the past week (Fuhrimann et al. 2022) (Tables 1, 2). However, several other measures of recent sleep problems assessed in this study were not associated with frequency of self-reported glyphosate use in the past week.

Otherwise, a low-quality ecological study that linked U.S. national-level data on glyphosate agricultural applications and various neurological outcomes, including hospital discharge rates, mortality rates, and childhood autism case counts, was identified but not considered further in our weight-of-evidence analysis due to its ecological design and other methodological weaknesses (Seneff et al. 2015) (Table 2 and Supplemental Table 1).

### Evidence synthesis

Among the 13 high- and moderate-quality studies overall, statistically significant associations were reported with five different neurological conditions, including one neurodegenerative outcome [age-related macular degeneration (Montgomery et al. 2017)], one neurobehavioral outcome [visual memory (Fuhrimann et al. 2021)], and three different other neurological outcomes (dream-enacting behaviors (Shrestha et al. 2018), olfactory impairment [Shrestha et al. 2021), and sleep problems (Fuhrimann et al. 2022)] (Table 1). That is, associations with specific neurological endpoints or even broader categories of endpoints were detected in only a single study each, all in moderate-quality studies with at least one important methodological limitation. The magnitude of association in these studies was generally modest, with relative risks ranging between 1.1 and 2.6 for age-related macular degeneration, dream-enacting behaviors, and olfactory impairment, up to 3.8 for one measure of sleep problems, and an average difference of 0.1 in visual memory as measured on a scale from 1 to 10. By definition, these solitary positive findings show no consistent association between glyphosate exposure and any neurological outcome in humans.

The only outcomes evaluated in more than one study population were Parkinson disease [studied twice in the AHS and once in the East Texas hospital-based case–control study (Dhillon et al. 2008; Kamel et al. 2007; Shrestha et al. 2020)] and depression [studied in male licensed pesticide applicators and their female spouses in the AHS (Beard et al. 2013, 2014)]. Across these four studies, neither neurological condition exhibited a statistically significant association with glyphosate exposure, and estimated relative risks were close to the null, ranging between 0.8 and 1.4.

Among the five high-quality studies and the eight moderate-quality studies, only six evaluated associations with quantitative or semi-quantitative levels of glyphosate use (Fuhrimann et al. 2021; Fuhrimann et al. 2022; Montgomery et al. 2017; Shrestha et al. 2020; Shrestha et al. 2021; Zhang et al. 2018),^1^ whereas the remaining seven studies evaluated only ever vs. never use of glyphosate (Beard et al. 2013, 2014; Dhillon et al. 2008; Juntarawijit et al. 2020; Kamel et al. 2007; Kamel et al. 2012; Shrestha et al. 2018). Ever vs. never use is an extremely limited classification of exposure that obscures a wide range of potential usage scenarios and cumulative doses. Adding the latter seven studies using ever vs. never exposure classification to the six studies with residential proximity-based or ecological exposure metrics (Caballero et al. 2018; Nevison 2014; Rull et al. 2006; Seneff et al. 2015; von Ehrenstein et al. 2019; Yang et al. 2014) and two other low-quality studies that assessed ever vs. never glyphosate use (Garry et al. 2002; Wechsler et al. 1991), three quarters of the available epidemiological studies of glyphosate and neurological outcomes (17 of 23, 74%) offer little to no information on the extent of any individual person’s exposure frequency or likely dose of glyphosate.

In light of the lack of an established statistical association between glyphosate and any neurological outcome or even any broader category of outcomes, application of the Hill guidelines to evaluate the weight of epidemiological evidence for causation (Hill 1965) is not warranted.

## Discussion

In this systematic literature review, we identified and considered 25 epidemiological studies of glyphosate and various neurological outcomes, including nine studies of neurodegenerative outcomes (two high quality, three moderate quality, and four low quality); five studies of neurobehavioral outcomes (two high quality, one moderate quality, and two low quality); six studies of neurodevelopmental outcomes (one moderate quality and five low quality); and five studies of other or mixed neurological outcomes (one high quality, three moderate quality, and one low quality). All of the high-quality studies, rated according to U.S. EPA OPP guidance for assessing methodological quality, found near-null associations between glyphosate use and neurological endpoints (namely, depression, Parkinson disease, and peripheral nerve conduction).

Eight moderate-quality studies variously found positive and null associations between glyphosate and a range of neurodegenerative, neurobehavioral, neurodevelopmental, and other neurological conditions, but no associations with a given endpoint were replicated in more than a single study population. Two of these studies found associations with self-reported dream-enacting behaviors and olfactory impairment, which may be considered early symptoms of neurodegenerative conditions, such as Parkinson disease (Shrestha et al. 2018; Shrestha et al. 2021), yet no association was found with Parkinson disease itself in moderate- or high-quality studies (Faria et al. 2014; Kamel et al. 2007; Shrestha et al. 2020). Any etiologic commonality of other neurological endpoints examined across studies is unknown, making it difficult to evaluate coherence of findings based on different outcomes.

The remaining 12 low-quality studies also reported a mixture of positive and null associations that are unlikely to reflect valid causal effects. Thus, the epidemiological literature on glyphosate and neurological conditions is sparse and mostly methodologically weak, and does not demonstrate an association between glyphosate exposure and risk of any specific neurological condition or category of neurological outcomes in humans.

As noted in the introduction, U.S. EPA (2017a) concluded in its draft human health risk assessment of glyphosate: “There was no evidence that glyphosate is neurotoxic.” Similarly, the Agency for Toxic Substances and Disease Registry (ATSDR) concluded in its Toxicological Profile for Glyphosate: “Neurological … endpoints have been evaluated, but do not appear to be particular targets of glyphosate toxicity” (ATSDR 2020). A recent systematic literature review of glyphosate neurotoxicity studies found no clear evidence of mammalian neurobehavioral, neuropathological, or neuropharmacological effects of glyphosate, albeit based on a limited evidence database (Moser et al. 2022, submitted). Thus, drawing from the toxicological literature, the biological plausibility of glyphosate neurotoxicity in humans is uncertain.

Across the body of epidemiological literature on glyphosate and neurological outcomes, the major methodological shortcoming relates to exposure assessment. Strictly following OPP guidance, none of the available epidemiological studies would be rated as having high-quality exposure assessment based on a metric with an “[a]ccurate and precise relationship with external exposure, internal dose, or target dose” (U.S. EPA 2016). As discussed earlier, the FFES biomonitoring study (Acquavella et al. 2004) showed that first-hand use of glyphosate produces a detectable, typically low short-term systemic dose, whereas glyphosate applicators’ household members who were not involved in the on-study application exhibit, with few exceptions, no detectable systemic dose. This conclusion is supported by other studies demonstrating, for example, no appreciable difference in urinary glyphosate level between members (including children) of farm and non-farm households or following agricultural applications of glyphosate (Curwin et al. 2007; Niemann et al. 2015; Solomon 2016). The lack of measurable urinary glyphosate in bystanders makes it implausible that indirect or remote exposure to glyphosate, for example, from residential proximity to glyphosate applications within 500 m, would result in any appreciable dose. Moreover, spray drift generally does not travel several hundred meters away from agricultural applications of herbicides (Bird et al. 1996; Carlsen et al. 2006). Therefore, any attempt to assess glyphosate exposure based on indirect measures, especially residential proximity, would produce largely, if not exclusively, false positive exposure classifications.

To date, no epidemiological studies have attempted to identify potential neurological effects related to residential (non-occupational) applications of glyphosate, either on home users or uninvolved family members. Such applications would almost always involve a much lower quantity of glyphosate than agricultural applications, and it seems unlikely that residential systemic doses from a glyphosate application would approach those seen in the FFES for applicators (median dose 0.0001 mg/kg) or for the few FFES family members who had detectable glyphosate doses (< 0.0001 mg/kg). Biomonitoring studies relative to residential applications, taking care to account for potential dietary exposure, are lacking, and would be a valuable addition to the literature.

Glyphosate usage patterns among applicators are also important to consider. From 1974 to at least the mid to late 1990s, glyphosate was typically applied to a given crop only once or twice per year (Benbrook 2016). Many applicators may use glyphosate only a handful of times in their lifetime, even in an occupational context. When conducting epidemiological research, investigators should consider the biological plausibility that a limited number of uses of glyphosate could cause a chronic neurodegenerative disorder, especially after a short putative latency period; or whether pre-conception parental exposure could plausibly cause a congenital central nervous system anomaly in offspring. Such an evaluation of biological plausibility would need to account for the facts that glyphosate is not appreciably metabolized, has a relatively short biological half-life measured in hours (Connolly et al. 2019), and is estimated to produce a systemic dose from an individual application that is 1000–10,000 times lower than the daily regulatory agency reference dose. These metabolic properties almost certainly contribute to the lack of detectable glyphosate dose from indirect exposure in biomonitoring studies. Such issues should be borne in mind when developing scientific hypotheses, designing exposure assessment methods, and conducting pre-study feasibility planning.

Outcome assessment is another important limitation in many of the available studies. Disparate diagnostic criteria can reduce comparability of results across studies, while also affecting internal study validity. Parkinson disease, for instance, was variously ascertained based on clinical examination by a neurologist specializing in movement disorders (the gold standard) (Dhillon et al. 2008); neurology clinic records or patient support groups (Wechsler et al. 1991); a self-reported doctor’s diagnosis (Kamel et al. 2007); a positive self-report confirmed by a movement disorder specialist based on clinical examination, questionnaire data, and/or medical records review (Shrestha et al. 2020); or death certificate (Caballero et al. 2018). Most studies relied to some extent on self-reported information on neurological conditions, some exclusively so (Beard et al. 2013, 2014; Faria et al. 2014; Fuhrimann et al. 2022; Garry et al. 2002; Kamel et al. 2007; Kim et al. 2013; Shrestha et al. 2018, 2021), thereby introducing a degree of misclassification that could have been extensive in some studies and, potentially, differential by exposure status.

Confounding remains a concern in virtually all studies, both for neurological disorders with many known risk factors (e.g., sleep problems, depression) and those with as yet few identified risk factors (e.g., Parkinson disease, ALS). None of the available studies had strictly high-quality confounder control based on “[g]ood control for important confounders relevant to [the] scientific question, and standard confounders” (U.S. EPA 2016). When infrequent use of a specific pesticide, such as glyphosate, takes place in a milieu of numerous other chronic exposures with potential biological activity, including other pesticides and aspects of an agricultural occupation or lifestyle, uncontrolled confounding and residual confounding (the latter due to insufficient adjustment for measured confounders) are difficult to eliminate.

In conclusion, based on our systematic review of the available epidemiological literature, we found no consistent evidence of a causal effect, or even a convincing statistical association, between glyphosate exposure and any neurological outcome in humans. To meaningfully advance our understanding of the potential neurotoxicity of glyphosate in humans, more epidemiological studies are needed in populations that have frequent direct exposure, validated outcome assessment, minimization of selection bias through achievement of high participation and retention rates, and rigorous statistical adjustment for potential confounding, especially for important personal factors and correlated pesticides. Past biomonitoring studies indicate that poor-quality exposure assessment or biologically implausible hypotheses involving ever-vs.-never use of glyphosate or indirect exposure pathways are unlikely to be informative. Nevertheless, additional biomonitoring studies could provide valuable insight into the validity of indirect exposure metrics based on geographic models or reported use by household members. Epidemiological research in this realm requires scientific prudence in determining which causal hypotheses may have merit, and whether the appropriate epidemiological data are available or attainable to investigate those hypotheses in a manner that yields valid and informative results.

## Supplementary Information

Below is the link to the electronic supplementary material.

Supplementary file1 (DOCX 179 KB)

## Footnotes

1 Fuhrimann et al. (2022) evaluated associations of sleep problems with number of days of glyphosate use in the past week, but did not report the results of this analysis.
