# Supplementary material for: Systematic literature review of the epidemiology of glyphosate and neurological outcomes
Source: Int Arch Occup Environ Health. 2022 May 23;96(1):1–26. doi: 10.1007/s00420-022-01878-0 (PMC9823069; doi:10.1007/s00420-022-01878-0)
Supplement: Supplementary file 1 — Supplementary file1 (DOCX 179 KB) [file 420_2022_1878_MOESM1_ESM.docx]

| **Author** | **Year** | **Country** | **Study Design** | **Study Population** | **Comparison Subjects** | **Exposure Assessment** | **Exposure Levels** | **Outcome** | **Outcome Assessment** | **Confounders Considered** | | **Statistical Approach** | | **Estimate of Association (95% CI)** | | **Funding Source** | |
| --- | --- | --- | --- | --- | --- | --- | --- | --- | --- | --- | --- | --- | --- | --- | --- | --- | --- |
| **Neurodegenerative outcomes** | | | |  |  |  |  |  |  |  | |  | |  | |  | |
| Andrew et al. | 2021 | U.S. | Healthcare-claims-based and population-based case-control | 26,199 ALS cases identified from SYMPHONY Integrated Dataverse de-identified healthcare claims database of patients aged ≥ 18 years with ≥ 6 months of prior enrollment, U.S. nationwide, 2013–2019  500 ALS cases identified from mortality records in New Hampshire (2009–2018), Vermont (2008–2016), and Ohio (2016–2019), with diagnosis or index year (not defined) on or after 2013 | 78,597 controls aged ≤ 80 years without ALS or other neurodegenerative diseases in SYMPHONY database, matched 3:1 to cases on age and sex  1,949 controls in New Hampshire/Vermont/Ohio identified from U.S. Postal Service Delivery Sequence files licensed to Marketing Systems Group, frequency matched to expected age and sex distribution of cases | Estimated annual county-level crop applications of glyphosate and 422 other pesticides, averaged over 2002–2012, based on pesticide-use estimates from U.S. Geological Survey and data on harvested crop acres from U.S. Department of Agriculture, imputed from neighboring counties for crops in counties with unreported pesticide data  Assigned to most recent residential region based on first 3 digits of ZIP code at diagnosis (SYMPHONY) or averaged over residential counties during 5 years prior to index date, with residential history identified from LexisNexis commercial financial marketing database (New Hampshire/Vermont/Ohio) | Glyphosate applied to crops per year: ≥ vs. < 9,090 kg/square mile (median) | ALS | In SYMPHONY database: ≥ 2 codes for ALS ≥ 3 months apart based on International Classification of Diseases, 9th or 10th revision, with ≥ 6 months' enrollment in database prior to first code, and age at first code ≥ 18 years  In New Hampshire/Vermont/Ohio: mortality records attributed to motor neuron disease using International Classification of Diseases, 10th revision code G12.2 | Adjusted: age, sex | | Multivariable logistic regression  Patients from SYMPHONY database randomly divided into "discovery cohort" of 500 geographic regions (based on first 3 digits of ZIP code) to identify associated pesticides, and "validation cohort" of remaining 363 geographic regions to test pesticides with false discovery rate < 0.1 | | **Odds ratio in SYMPHONY validation dataset = 1.29 (1.19, 1.39)  Odds ratio in New Hampshire/Vermont/Ohio dataset = 2.02 (1.64, 2.49)** | | Mitsubishi Tanabe Pharma America; Agency for Toxic Substances and Disease Registry | |
| Caballero et al. | 2018 | U.S. | Population-based case-case | 659 Parkinson disease deaths at ages ≤ 75 y ("premature deaths") among residents of Washington State, 2011–2015 | 3,932 Parkinson disease deaths at ages > 75 y among residents of Washington State, 2011–2015 | Residential proximity (yes or no) using address at death within 1,000 m of agricultural land likely to have been treated with glyphosate, linked to state cropland data from US. Department of Agriculture and estimated pesticide use by type of crop according to U.S. Geological Survey; well water proximity (yes or no) within 500 m of agricultural land likely to have been treated with glyphosate, 3 other specific pesticides, or total pesticides based on residential wells within 250 m of address of death, linked to same crop-pesticide matrix; or occupational exposure to agricultural chemicals (yes or no) based on "Agriculture, Forestry, Fishing, and Hunting" industry and "Crop Production" occupation listed on death certificate | Residential proximity (within 1,000 m) or well water proximity (within 500 m) at death to agricultural crops considered likely to have been treated with glyphosate (659 deaths) | Parkinson disease death at ≤ 74 y | Death from Parkinson disease as direct or underlying cause, identified based on International Classification of Diseases code from Washington State Department of Health death certificates | Adjusted: sex, race, marital status, education | | Multivariable logistic regression | | **Odds ratio, unadjusted = 1.30 (1.04, 1.62)  Odds ratio, adjusted = 1.33 (1.06, 1.67)** | | None; authors funded by Health Equity Research Center, Washington State University; open-source publication funded by Asprey Center for Collaborative Approaches to Science and Vassar College Academic Enrichment Fund | |
| Wan & Lin | 2016 | U.S. | Ecological | Annual county-level incidence rates of Parkinson disease in Nebraska, based on 6,557 Parkinson disease cases from the Nebraska Parkinson Disease Registry, 1997–2008 | Spatial patterns by county | Estimated application of glyphosate and 19 other pesticides to corn, soybeans, small grains, alfalfa, and sorghum (98% of crops in Nebraska), based on annual agricultural pesticide usage data in 2005 from U.S. Geological Survey, combined with surveys of farmers' pesticide usage by crop type and irrigation status in 1982–1987 from the University of Nebraska-Lincoln, and in 1992 and 1997 from the National Center for Food and Agricultural Policy, linked to land-use data in 2005 from the Center for Advanced Land Management Information Technologies at the University of Nebraska-Lincoln | Not applicable | Parkinson disease | Annual county-level age-adjusted incidence rate of Parkinson disease, ascertained by Nebraska Department of Health and Human Services through mandatory physician reporting of new diagnoses within 60 days, and semiannual mandatory pharmacy reporting of patients who received one or more anti-Parkinson-disease medications, traced back to physicians to confirm diagnosis and date and address of diagnosis as accurately as possible | Adjusted, joint model: crop density, county-level use of atrazine, bromoxynil, alachlor, and metribuzin  Adjusted, single-pesticide model: county-level poverty rate, crop density, and percent of farmers | | Multivariable linear regression | | Joint model: β coefficient for quartile 2 vs. 1 = -0.041, p ≥ 0.05 β coefficient for quartile 3 vs. 1 = 0.083, p ≥ 0.05 β coefficient for quartile 4 vs. 1 = 0.135, p ≥ 0.05  Adjusted *R*^2^ = 0.32  Single-pesticide model: β coefficient for quartile 2 vs. 1 = 0.118, p ≥ 0.05 **β coefficient for quartile 3 vs. 1 = 0.279, p < 0.05 β coefficient for quartile 4 vs. 1 = 0.313, p < 0.05** Adjusted *R*^2^ = 0.240 | | None | |
| Wechsler et al. | 1991 | U.S. | Hospital-based case-control | 34 Parkinson disease patients aged ≥ 40 y identified from University Hospital Neurology Clinic at University of Washington, and Seattle area Parkinson disease support groups; years NR, overall participation 49% (59 of 121 including cases and controls) | 22 controls aged ≥ 40 y with neurological disorders other than Parkinson disease and dementia, identified from University Hospital Neurology Clinic at University of Washington; participation 49% (59 of 121 including cases and controls) | Self-reported occupational and home use of Round-Up and ≥ 8 other specific pesticides, as well as various occupations and metals, ascertained by pre-tested, structured, mailed questionnaire, with follow-up reminder telephone call to Neurology Clinic subjects if questionnaire was not yet received | Use of Round-Up as a "home pesticide" (14 cases, 9 controls) | Parkinson disease | Parkinson disease identified from neurology clinic or patient support groups, diagnosis not reported as confirmed | None | | "Frequency analysis" (chi-square) | | Odds ratio = (14*13)/(20*9) = 1.01 (0.34, 3.01) | | NR | |
| **Neurobehavioral outcomes** | | | |  |  |  |  |  |  |  | |  | |  | |  | |
| Faria et al. | 2014 | Brazil | Cross-sectional | 2,400 tobacco farmers aged ≥ 18 years engaged in farming activities for ≥ 15 hours per week, identified from farms randomly selected from tobacco sales invoices issued by government mandate in 2009, interviewed at their farms during 8-week fieldwork period during harvest, São Lourenço do Sul-RS, southern Brazil, January and February 2011; 92.3% participation  288 (12.0%) with minor psychiatric disorders | Subjects without glyphosate exposure | Self-reported use of glyphosate and 55 other pesticides on the farm (not necessarily personally applied) in the past year, prompted by cards with photographs and trade names | Glyphosate used on farm in the past year (1,565 subjects) | Minor psychiatric disorders | Non-psychotic mental disorders assessed using Self-Reporting Questionnaire 20 items, developed to screen for common mental disorders in primary health care, including somatic factors (e.g., headaches, appetite, digestion, sleep), depressive/anxiety symptoms (e.g., feeling frightened, unhappy, or worthless; crying), and cognitive/decreased energy factors (e.g., not being able to concentrate or make decisions, suffering at work, being unable to enjoy daily activities); classified as "positive" based on ≥ 6 alterations in men or ≥ 8 in women (≥ 7 or ≥ 8, respectively, in sensitivity analyses) | Adjusted: age, sex, annual tobacco production, employment status, difficulty in paying debts, alcohol consumption, religious activity, farming workday hours, strenuous work  Considered: vehicle ownership, percentage of income arising from tobacco, family history of suicide, smoking, intense working pace, green tobacco sickness, chronic low back pain, pesticide poisoning | | Hierarchical multivariable Poisson regression with robust variance estimation | | Prevalence ratio, adjusted = 0.81 (0.65, 1.01)  **Prevalence ratio, unadjusted = 0.71 (0.57, 0.88)**  Prevalence of minor psychiatric disorders on farms not using glyphosate = 14.8% Prevalence of minor psychiatric disorders on farms using glyphosate = 10.5% Wald p = 0.002 | | National Council for Scientific and Technological Development; Research Support Foundation of the Rio Grande do Sul State | |
| Kim et al. | 2013 | South Korea | Cross-sectional | 1,895 male farmers residing in rural areas, identified from nationwide sampling survey using stratified multi-stage probability sampling methods, South Korea, February and March 2011; 94.8% participation with non-missing data | Subjects without glyphosate exposure | Self-reported acute occupational pesticide poisoning in 2010, identified based on reporting any of 21 signs and symptoms (nausea, vomiting, diarrhea, sore throat, runny nose, dyspnea, headache, dizziness, hyperactivity, profuse sweating, blurred vision, paresthesia, slurred speech, paralysis, chest pain, syncope, muscle weakness, skin irritation, eye irritation, lacrimation, fatigue) within 48 hours of occupational pesticide use, with further inquiry about pesticide products (including glyphosate) associated with poisoning | Acute occupational poisoning by glyphosate in prior year (2 subjects with depressive symptoms, 23 subjects without depressive symptoms) | Depressive symptoms | Self-reported depressive symptoms assessed by Korean version of the Geriatric Depression Screening Scale short form, chosen as an "adequate screening instrument for detecting major depressive episodes" as defined by criteria from Diagnostic and Statistical Manual of Mental Disorders, 4th Edition, for outpatients aged ≥ 60 years; based on yes/no questions on 15 specific depressive symptoms during the past week, with total scores ≥ 8 classified as having depressive symptoms | Adjusted: age, income, marital status, smoking, physician-diagnosed comorbidity, perceived health status  Considered: education, farm size, type of farming, application methods, use of personal protective equipment, safety behavior when applying pesticides, alcohol drinking, obesity | | Multivariable logistic regression | | Odds ratio, adjusted = 0.49 (0.11, 2.13)  Odds ratio, unadjusted = 0.64 (0.14, 2.75) | | National Research Foundation of Korea; Cooperative Research Program for Agriculture Science and Technology Development, Rural Development Administration, Republic of Korea | |
| **Neurodevelopmental outcomes** | | | |  |  |  |  |  |  |  | |  | |  | |  | |
| Garry et al. | 2002 | U.S. | Cross-sectional | 1,532 children born to 695 families of male licensed pesticide applicators (98% participation) living in Red River Valley, Minnesota, 1991–1996  14 cases with congenital central nervous system anomaly, 2 autism cases, 14 ADD/ADHD cases | Control children without congenital anomalies, without autism, or without ADD/ADHD | Self-reported ever use of phosphonamino herbicides (glyphosate, Roundup) and other specific pesticides (number NR) in 15 pesticide classes reported by telephone interview, with reinterview by written questionnaire ~6 months later, surveyed in 1997–1998; phone survey data used in analysis in absence of written questionnaire data | Parental ever use of glyphosate (6 of 14 children with ADD/ADHD) | Congenital central nervous system anomaly, autism, ADD/ADHD | Congenital central nervous system anomaly, autism, or ADD/ADHD self-reported by written questionnaire, with confirmation of congenital anomalies (not autism or ADD/ADHD) based on birth certificate and medical records if possible | Adjusted: NR  Considered: mother's age, smoking status, alcohol use, season of conception, chronic diseases such as diabetes, pharmacologically treated hypertension, arthritis, occupations other than agriculture, residence at a rural site or on a farm during childhood | | "Regression analysis" | | **Odds ratio, ADD/ADHD = 3.6 (1.35, 9.65)** Congenital central nervous system anomaly: no association (NR)  Autism: no association (NR) | | U.S. National Institute of Environmental Health Sciences, people of the State of Minnesota | |
| Nevison | 2014 | U.S. | Ecological | U.S. children with autism aged 6–17 y in 1991–2010 + age 5 y in 2000–2010 from Individuals with Disabilities Education Act database, and California children with autism aged ≥ 5 y as of 2002 and aged 5 y in 1995–2006 from California Department of Developmental Services | Temporal trends by birth year | Estimated annual application of glyphosate to genetically modified corn and soy crops (in units of 1,000 tons/y), based on U.S. Department of Agriculture state-level data on crop acreage, percentage of corn and soybean crops that are genetically engineered, and glyphosate application rates per acre; also evaluated trends in various other pesticides, chemicals, and substances | Not applicable | Autism | Autism prevalence by year of birth ascertained based on counts from Individuals with Disabilities Education Act database and California Department of Developmental Services, divided by statewide grade-specific public school populations from National Center for Education Statistics annual reports in 1991–2010, with denominator adjusted upward by 14% to account for private school populations | None | | Correlation coefficient | | **Correlation ρ, California 2002 = 0.75, p < 0.05  Correlation ρ, U.S. = 0.92, p < 0.05** | | None; study conducted by author as volunteer for SafeMinds Environmental Research Committee | |
| Rull et al. | 2006 | U.S. | Population-based case-control | 731 neural tube defect cases (including elective terminations) identified by California Birth Defects Monitoring Program, sampled from singleton liveborn infants and fetal deaths delivered 1987–1988 to women in most California counties (84% participation), and 1989–1991 in all California counties except Los Angeles, Ventura, and Riverside (88% participation) | 940 controls without neural tube defects sampled from same sources (74% and 88% participation) | Residential proximity (yes or no) using self-reported address(es) during 2-month periconceptional period (geocoded for 92% of cases, 94% of controls) within 1,000 m of agricultural applications of glyphosate and 58 other specific pesticides, identified from pesticide use reports submitted to California Department of Pesticide Regulation in 1986–1991, linked to countywide land-use surveys from California Department of Water Resources conducted every 7–10 y, based on survey closest to year of conception | Residential proximity during periconception period within 1,000 m of agricultural crops treated with glyphosate (45 cases, 33 controls) | Neural tube defects | Confirmed diagnosis of anencephaly, spina bifida cystica, craniorachischisis, or iniencephaly, ascertained from California Birth Defects Monitoring Program | Adjusted: study period, maternal race/ethnicity, education, periconceptional smoking, vitamin use, all other pesticides and physicochemical categories of pesticides in hierarchical model | | Two-stage hierarchical multivariable logistic regression, with all pesticides and covariates of interest in conventional dichotomous or polytomous stage 1, and pesticide-specific maximum-likelihood coefficients from first-stage model regressed on linear model with 12 dichotomous indicator variables of physicochemical categories containing ≥ 2 pesticides each in stage 2 | | Odds ratio, single-pesticide model = 1.5 (1.0, 2.4)  Odds ratio, multiple-pesticide model = 1.5 (0.8, 2.9)  Odds ratio, hierarchical multiple-pesticide model = 1.4 (0.8, 2.5)  "We also observed subtype associations between anencephaly and … glyphosate" (results NR) | | NR | |
| von Ehrenstein et al. | 2019 | U.S. | Population-based case-control | 2,961 autism spectrum disorder cases (445 with intellectual disability comorbidity, 2,516 without) identified from registry maintained at California Department of Developmental Services, including children born in eight major agricultural counties of Central Valley, California, in 1998–2010, based on probabilistic linkage to California birth records at Office of Vital Statistics; linkage rate 86.3% | 35,370 controls without autism spectrum disorder randomly matched 10:1 to cases by birth year and sex, identified from California birth records in same counties and birth years | Residential proximity (yes or no) using birth address within 2,000 or 2,500 m of agricultural applications of glyphosate and 10 other specific pesticides identified from pesticide use reports submitted to California Department of Pesticide Regulation, linked to countywide land-use surveys from California Department of Water Resources, with monthly average pounds applied per acre generated by geographic information system and applied to 3-month pre-conception period, gestational period by month, and first year of life | Residential proximity at birth within 2,000 m (2,500 m in sensitivity analyses) of agricultural crops treated with glyphosate in 3 months before pregnancy, during pregnancy, or in first year of life (2,293 cases, 26,660 controls during pregnancy; 2,256 cases, 26,390 controls during first year of life) | Autism spectrum disorder, with or without intellectual disability | Autism spectrum disorder ascertained from California Department of Developmental Services through contracted regional centers, following diagnostic criteria of the *Diagnostic and Statistical Manual of Mental Disorders*, fourth edition (DSM-IV); comorbid intellectual disability diagnosed according to DSM-IV criteria corresponding to International Classification of Diseases, 9th revision, code for "mental retardation" | Adjusted: year of birth, sex, maternal race/ethnicity, maternal age, maternal education, modeled ambient nitrogen oxides concentration within 1,500 m of birth address; in some models: residential proximity during three periods (periconception, pregnancy, first year of life), residential proximity to up to 2 other associated pesticides  Considered: maternal birthplace, urban vs. rural residence, census-based socioeconomic status, source of payment for delivery, preterm birth | | Multivariable logistic regression; semi-Bayesian hierarchical models in sensitivity analyses (not shown) | | Autism spectrum disorder with or without intellectual disability:  Odds ratio, 3 months before pregnancy, adjusted for other periods = 0.97 (0.87, 1.08) Odds ratio, during pregnancy, adjusted for other periods = 1.11 (0.96, 1.28) Odds ratio, first year, adjusted for other periods = 1.09 (0.94, 1.26)  **Odds ratio, during pregnancy = 1.16 (1.06, 1.27)** Odds ratio, during pregnancy, adjusted for 10 other pesticides = 1.12 (0.99, 1.27) **Odds ratio, during pregnancy, adjusted for permethrin = 1.14 (1.03, 1.26)** Odds ratio, during pregnancy, adjusted for permethrin and chlorpyrifos = 1.09 (0.98, 1.23) **Odds ratio, during pregnancy, adjusted for permethrin and myclobutanil = 1.16 (1.04, 1.29) Odds ratio, during pregnancy, adjusted for permethrin and methyl bromide = 1.14 (1.04, 1.26)**  **Odds ratio, first year = 1.15 (1.05, 1.26) Odds ratio, first year, adjusted for 10 other pesticides = 1.17 (1.04, 1.32)** | | U.S. National Institute of Environmental Health Sciences | |
| von Ehrenstein et al. (continued) | 2019 | " | " | " | " | " | " | " | " | " | | " | | Autism spectrum disorder with intellectual disability:  Odds ratio, 3 months before pregnancy, adjusted for other periods = 0.96 (0.74, 1.26) Odds ratio, during pregnancy, adjusted for other periods = 0.96 (0.66, 1.40) **Odds ratio, first year, adjusted for other periods = 1.60 (1.09, 2.34)  Odds ratio, during pregnancy = 1.33 (1.05, 1.69)** Odds ratio, during pregnancy, adjusted for 10 other pesticides = 1.12 (0.82, 1.53) Odds ratio, during pregnancy, adjusted for permethrin = 1.17 (0.91, 1.50) Odds ratio, during pregnancy, adjusted for permethrin and chlorpyrifos = 1.12 (0.84, 1.51) Odds ratio, during pregnancy, adjusted for permethrin and myclobutanil = 1.08 (0.82, 1.43) Odds ratio, during pregnancy, adjusted for permethrin and methyl bromide = 1.12 (0.86, 1.45)  **Odds ratio, first year = 1.51 (1.18, 1.92)** Odds ratio, first year, adjusted for 10 other pesticides = 1.37 (0.99, 1.89) **Odds ratio, first year, adjusted for permethrin = 1.34 (1.03, 1.74)** Odds ratio, first year, adjusted for permethrin and chlorpyrifos = 1.33 (0.98, 1.81) Odds ratio, first year, adjusted for permethrin and myclobutanil = 1.31 (0.99, 1.74) Odds ratio, first year, adjusted for permethrin and methyl bromide = 1.29 (0.98, 1.68) | | " | |
| Yang et al. | 2014 | U.S. | Population-based case-control | 73 anencephaly cases, 123 spina bifida cases, 277 cleft lip cases with or without cleft palate, and 117 cleft palate alone cases delivered in 8 counties of San Joaquin Valley, California, 1997–2006, identified by California Birth Defects Monitoring Program through active surveillance at all hospitals with obstetric or pediatric services, cytogenetic laboratories, and all clinical genetics prenatal and postnatal outpatient services, excluding cases with single-gene conditions, chromosomal abnormalities, or identifiable syndromes, and excluding mothers with pregestational diabetes; 71% participation | 785 nonmalformed, live-born controls randomly selected from same birth hospitals as cases, excluding mothers with pregestational diabetes; 69% participation | Residential proximity (yes or no) using self-reported address(es) during 3-month periconceptional period (geocoded for 82% of cases, 83% of controls) with 500 m of agricultural applications of glyphosate and other specific pesticides (number NR) in 33 pesticide groups, and identified from pesticide use reports submitted to California Department of Pesticide Regulation in 1997–2006, linked to countywide land-use surveys from California Department of Water Resources conducted every 5–7 y | Residential proximity during periconception period within 500 m of agricultural crops treated with glyphosate (12 anencephaly cases, 21 spina bifida cases, 57 cleft lip with or without cleft palate cases, 23 cleft palate alone cases, 166 controls) | Neural tube defects: anencephaly, spina bifida, cleft lip with or without cleft palate, cleft palate alone | Diagnosis of anencephaly, spina bifida, cleft lip with or without cleft palate, or cleft palate alone confirmed by clinical geneticists based on clinical, surgical, or autopsy reports | Adjusted: maternal race/ethnicity, education, pre-pregnancy body mass index, parity, folic acid supplement use, periconceptional smoking; infant sex (stratified) for cleft lip and cleft palate | | Multivariable logistic regression | | Odds ratio for anencephaly = 0.8 (0.4, 1.6)  Odds ratio for spina bifida = 0.8 (0.5, 1.4)  Odds ratio for cleft lip with or without cleft palate = 0.9 (0.6, 1.3)  Odds ratio for cleft palate alone = 0.9 (0.5, 1.5) | | Gerber Foundation, U.S. Centers for Disease Control and Prevention | |
| **Other/mixed neurological outcomes** | | | |  |  |  |  |  |  |  | |  | |  | |  | |
| Seneff et al. | 2015 | U.S. | Ecological | Annual disease-specific U.S. hospital discharge rates, based on National Hospital Discharge Survey data from U.S. Centers for Disease Control and Prevention, 1998–2010  Age-adjusted cause-specific mortality rates, based on data from U.S. Centers for Disease Control and Prevention mortality files  Counts of U.S. children aged 6 y diagnosed with autism, based on U.S. Department of Education data collected under Individuals with Disabilities Education Act, 1995–2010 | Temporal trends by year | Estimated annual application of glyphosate to corn and soy crops (in units of pounds/y), based on U.S. Department of Agriculture state-level data on total glyphosate applied to corn and soy crops and total acreage planted in surveyed U.S. states, 1990–2010 | Not applicable | Hospital discharges: sleep disorder, autism, ADHD, anxiety, schizophrenia, dementia  Deaths: suicide by overdose of prescription drugs, Alzheimer disease, dementia  Childhood autism | National annual hospital discharge rates per 1,000 to 100,000 coded based on International Classification of Diseases diagnosis codes, including any of up to seven discharge codes; list of outcomes analyzed NR  National annual mortality rates per 100,000 coded based on International Classification of Diseases diagnosis codes; list of outcomes analyzed NR  National annual autism counts based Individuals with Disabilities Education Act database | None for hospital discharge data  Age for autism and mortality data | | Correlation coefficient | | **Sleep disorder discharge correlation ρ = 0.99, p ≤ 7.7 × 10^-6^  Autism discharge correlation ρ = 0.98, p ≤ 9.6 × 10^-6^  ADHD discharge correlation ρ = 0.95, p ≤ 3.6 × 10^-5^  Anxiety discharge correlation ρ = 0.95, p ≤ 3.2 × 10^-5^  Schizophrenia discharge correlation ρ = 0.88, p ≤ 2.5 × 10^-4^  Dementia discharge correlation ρ = 0.93, p ≤ 5.8 × 10^-5^  Suicide by overdose of prescription drugs correlation ρ = 0.95, p ≤ 3.0 × 10^-8^  Alzheimer disease death correlation ρ = 0.96, p ≤ 1.7 × 10^-8^  Senile dementia death correlation ρ = 0.99, p ≤ 2.3 × 10-9  Childhood autism correlation ρ = 0.997, p ≤ 2.4 × 10^-7^** | | Quanta Computer, Inc., Taiwan | |
| ADD/ADHD: attention deficit disorder/attention deficit hyperactivity disorder; ALS: amyotrophic lateral sclerosis; CI: confidence interval; NR: not reported | | | | | | | | | | |  | |  | |  | |  |
| Statistically significant associations are shown in bold font. | | | | | | |  |  |  |  | |  | |  | |  | |

**
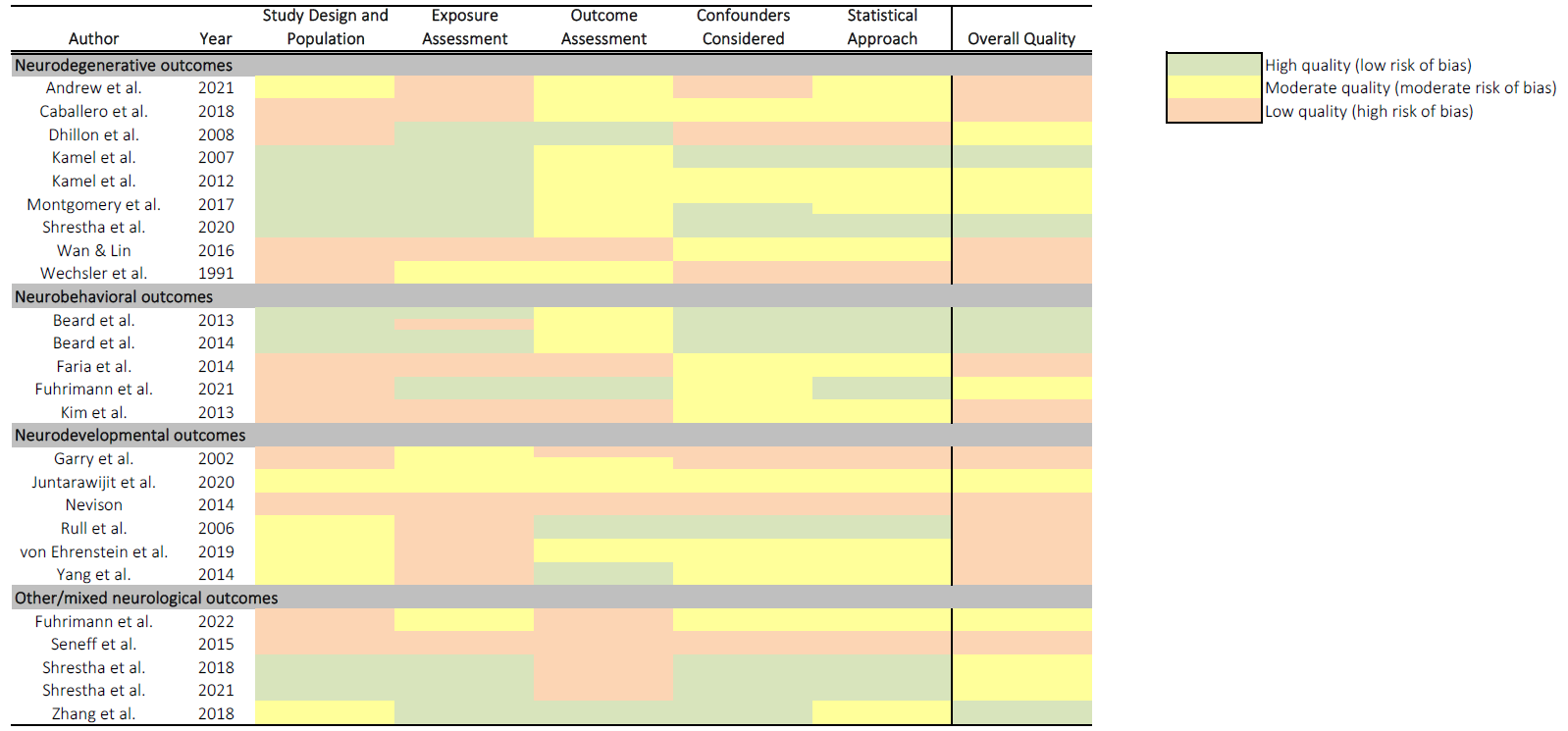
**
